# Preoperative gut microbiota combined with machine learning for predicting post-hepatectomy liver failure in HBV-related hepatocellular carcinoma: a pilot study

**DOI:** 10.3389/fcimb.2026.1877450

**Published:** 2026-08-06

**Authors:** Yu-Chong Peng, Jing-Xuan Xu, Hai-Yan Lu, Yu-Miao Bi, Lu-Nan Qi, Ying-Guo Chen, Jian-Hong Zhong, Andrea Schlegel, Le-Qun Li

**Affiliations:** 1 Department of General Surgery, Chongqing Traditional Chinese Medicine Hospital, Chongqing, China; 2 Chongqing University of Chinese Medicine, Chongqing, China; 3 Department of Breast and Thyroid Surgery, The First Affiliated Hospital of Chongqing Medical University, Chongqing, China; 4 Department of Hepatobiliary Surgery, Guangxi Medical University Cancer Hospital, Nanning, China; 5 Guangxi Liver Cancer Diagnosis and Treatment Engineering and Technology Research Center, Nanning, China; 6 Department of Inflammation and Immunity, Lerner Research Institute, Cleveland Clinic, Cleveland, OH, United States; 7 Transplantation Center, Cleveland Clinic, Cleveland, OH, United States

**Keywords:** 16S rRNA sequencing, gut microbiome, hepatitis B virus-related hepatocellular carcinoma, machine learning models, microbiome functions, post-hepatectomy liver failure

## Abstract

**Objective:**

This study investigated the association between preoperative gut microbiota (GM) profiles and post-hepatectomy liver failure (PHLF) in patients with newly diagnosed HBV-related hepatocellular carcinoma (HBV-HCC), aiming to explore non-invasive, modifiable GM biomarkers for perioperative risk stratification.

**Methods:**

A total of 233 fecal samples underwent 16S rRNA sequencing and were stratified into three distinct sets: a training set (*n* = 179), an internal validation set (*n* = 32), and an independent external validation set (*n* = 22). Preoperative GM features were compared between patients with and without PHLF to identify diagnostic biomarkers and construct machine learning-driven risk models (XGBoost, Random Forest [RF], and Gradient Boosting Machine [GBM]).

**Results:**

Significant β-diversity differences (Weighted UniFrac) were observed between the groups, identifying 11 differential genera; PHLF patients exhibited a characteristic enrichment of Faecalibacterium, and Blautia, alongside a profound depletion of Bacteroides. Functional inferences revealed distinct metabolic remodeling in the PHLF group, characterized by the upregulation of amino acid biosynthesis (specifically lysine and arginine) and nucleoside catabolism, coupled with a prominent downregulation of the hepatic-interactive urea cycle, central carbon metabolism (glycolysis and tricarboxylic acid [TCA] cycle), and short-chain fatty acid [SCFA] (butyrate) metabolism. In the independent external validation set, the XGBoost, RF, and GBM models achieved areas under the receiver operating characteristic curve (AUCs) of 78.12%, 71.88%, and 63.54%, respectively. Importantly, the models showed potent exclusionary performance, with specificities of 81.25% for XGBoost and RF versus 72.22% for GBM, while all three maintained a stable negative predictive value (NPV) of 81.25% and exceptionally stable negative F1-scores (F1- negative).Decision curve analysis (DCA) confirmed notable clinical net benefit of all models.

**Conclusion:**

We are among the first to characterize distinct GM signatures in PHLF patients with cross-geographic reproducibility. GM demonstrates strong potential as a non-invasive tool for preoperative risk stratification targeting the primary endpoint of PHLF. Characterized by high specificity and potent exclusionary capacity, this signature establishes a clinically robust framework for accurate low-risk patient identification and triage, thereby optimizing perioperative management.

## 1 Introduction

Hepatocellular carcinoma (HCC) ranks as the second most common cause of cancer-related deaths globally. Its incidence has steadily increased, particularly in China. HCC arises mainly from chronic liver conditions and is closely associated with hepatitis B or C viral (HBV, HCV) infections, cirrhosis, alcohol use, non-alcoholic steatohepatitis, and aflatoxin; HBV infection is particularly prevalent in China (Dong et al., 2020; Wang et al., 2023). Currently, China leads the world in the number of patients with liver cancer and distinguishes treatments targeting different stages of HCC. These treatments include hepatectomy, liver transplantation, transcatheter arterial chemoembolization, ablation, radiotherapy, tyrosine kinase inhibitors, and newly developed immune checkpoint inhibitors, serving both curative and palliative purposes (Kudo, 2020; Mohr et al., 2021). In recent years, these diverse treatment modalities have achieved some success in the treatment of liver cancer; however, overall, treatment remains unsatisfactory. Currently, hepatectomy remains the primary curative, radical treatment option for individuals with resectable HCC (Huang et al., 2023). While refinements in surgical modalities have progressively mitigated the mortality and complications (Peng et al., 2023), post-hepatectomy liver failure (PHLF) persists as the most catastrophic complication and the definitive bottleneck limiting surgical efficacy. PHLF drastically escalates perioperative mortality rates and imposes a staggering healthcare burden on medical systems (Bartholomä et al., 2025). underscoring an urgent clinical mandate for accurate, non-invasive preoperative risk prediction. The International Study Group of Liver Surgery (ISGLS) (Ye et al., 2020) has redefined PHLF. Given its high incidence and resource dependency, accurately and objectively identifying patients without PHLF across multiple dimensions provides more meaningful data for subsequent diagnostics and interventions, offering substantial clinical benefits that ultimately enhance overall patient outcomes and system efficiency.

In recent years, with the exploration of gut-related high-throughput data at the molecular level and the connection between the gut microbiome (GM) and disease, the GM, as a “hidden organ” and “second genome” (Gu et al., 2023), significantly impacting human health and various diseases. The GM strongly affects metabolism, the immune system, and the endocrine system, both at the local mucosal and systemic host levels (Zheng et al., 2020). Furthermore, convincing results have been obtained for its involvement in neuropsychiatric disorders (Fujita et al., 2023), type 2 diabetes (Chen et al., 2023), lung cancer (Seelbinder et al., 2023), colorectal cancer (Spatz et al., 2023), and liver cancer (Peng et al., 2023). In general, the GM has been shown to consistently reflect the close interplay between the host’s ecology and metabolism in a wide range of liver diseases (Kasztelan-Szczerbinska et al., 2023), suggesting that human diseases are closely related to the dynamics of the GM. The GM significantly influences liver injury development and progression. Importantly, a significant enrichment of *Bacteroides* has been cross-validated in the fecal microbiota of patients with metabolic dysfunction-associated steatotic liver disease, the downregulated expression of pivotal tight junction proteins compromises intestinal epithelial barrier integrity and heightens intestinal permeability, facilitating the transport of flora and endotoxins. This process activates liver immune cells, triggering inflammation. Inflammation exacerbates fat degeneration and induces changes in bile and amino acid metabolism, which can damage the liver (Pabst et al., 2023; Wang et al., 2026). During the progressive transition from chronic HBV infection to liver fibrosis, cirrhosis, and ultimately HCC, distinct structural shifts are undergone by the GM (Sehgal et al., 2020). This pathologically driven progression is consistently characterized by a stepwise depletion of homeostatic, butyrate-producing taxa and a concurrent enrichment of opportunistic pathogens. Translocated endotoxins and lipopolysaccharides (LPS) subsequently activate hepatic immune cells, triggering sustained portal hypertension and liver cell damage. while (Yang et al., 2020; Pabst et al., 2023). From a mechanistic perspective, the integrity of the intestinal barrier is undermined by the downregulation of tight junction proteins (Pabst et al., 2023). In addition, correlations have been identified between specific microbial signatures and aggressive tumor features, including microvascular invasion and related amino acid metabolic impairment (Peng et al., 2023). These findings provide additional evidence of the strong connection between the GM and the liver.

GM signatures, as noninvasive biomarkers, have exciting diagnostic and predictive potential for liver-related diseases. Conventionally, preoperative PHLF risk assessment relies on traditional clinical scoring systems or indocyanine green clearance tests (ICG), yet these methods exhibit suboptimal predictive accuracy and operational inefficiency. This diagnostic gap arises as early, subtle gut–liver axis metabolic and inflammatory disturbances escape detection by standard blood or volumetric assays, leaving a critical predictive blind spot pre-surgical stress. The research by Zheng et al. indicates that GM can affect multiple metabolic pathways, hasten the progression of diseases, and be a significant noninvasive biological marker for liver cancer diagnosis (Ren et al., 2019). Oh et al. used a RF model to study characteristic GMs in cohorts of external validation studies to anticipate liver cirrhosis diagnosis (Oh et al., 2020). However, the preoperative microbial signatures associated with liver failure following hepatectomy remain entirely uncharacterized to date.

To develop a preoperative risk-stratification framework for resectable HBV-HCC, we initially contrasted preoperative GM profiles between patients developing PHLF and non-PHLF. Following this initial characterization, distinct microbial biomarkers associated with liver failure were identified at the amplicon sequence variant (ASV) resolution. Beyond taxonomic identification, potential metabolic pathways linking gut dysbiosis to PHLF were further evaluated through functional prediction analysis. Finally, a GM-driven preoperative risk-stratification model for PHLF was developed and externally validated. Clinically, this framework functions as a high-specificity exclusionary screening tool, allowing low-risk surgical candidates to be accurately identified.

## 2 Methods

### 2.1 Study design and sample collection

From September 2020 to June 2024, 16S rRNA sequencing data were collected from two regions (Guangxi and Chongqing). The training set (PHLF group = 63, without PHLF group = 116) was from the Second Liver Ward, Guangxi, followed by an internal validation set (PHLF group = 11, without PHLF group = 21) from the First Liver Ward. An independent external validation set (PHLF group = 6, without PHLF group = 16) was obtained from Chongqing. Patients without PHLF were referred to as non-PHLF. According to the ISGLS judgment criteria, the group with PHLF was labeled preoperatively as bo.PHLF, and the group without PHLF was labeled nbo.PHLF. Briefly, PHLF is defined by an increasing serum bilirubin concentration and increasing international normalized ratio (INR) on or after postoperative day 5 compared with the values of the previous day (Ye et al., 2020).

Chronic HBV infection was strictly defined as documented serum hepatitis B surface antigen (HBsAg) positivity spanning at least 6 months prior to surgery, in accordance with the European Association for the Study of the Liver (EASL) and American Association for the Study of Liver Diseases (AASLD) guidelines. Patients were enrolled irrespective of their preoperative serum HBV-DNA titers, Our study focused on patients who were first diagnosed with HBV-HCC and had not been treated with any anti-tumor therapies before surgery, specifically those not on an immunosuppressive regimen, and had only undergone tumor surgery previously. All participants lacked a history of drug allergies, alcohol addiction, asthma, non-alcoholic fatty liver disease (NAFLD), gastrointestinal diseases, inflammatory bowel diseases, chronic infectious diseases, diabetes, or malignancies aside from those of the liver. Prior to surgery, patients did not take any medication, probiotics, or antibiotics for a period of 4 weeks. Prophylactic antibiotics were administered for 48 h after surgery, starting at the same initial time. Collected variables included patient demographics [age, body mass index (BMI), smoking, drinking] and clinical/oncological parameters [Child–Pugh grade, Barcelona Clinic Liver Cancer (BCLC) stage, tumor size, portal hypertension, computed tomography (CT) cirrhotic nodules, ascites, and liver resection >3 segments]. Laboratory assessments spanned routine hematology (platelets, hemoglobin), coagulation (prothrombin time, INR), lipid metabolic markers (total cholesterol), liver biochemistry [bilirubin, albumin, prealbumin (PA), total protein, alanine aminotransferase (ALT), aspartate aminotransferase (AST), total bile acid], and viral/tumor-specific indices [HBV-DNA, HBsAg, alpha-fetoprotein (AFP), and ICG excretion]. Fecal specimens were collected from 8:00 to 9:00 AM the day before surgery using sterile, DNA-free containers. Rigorous bedside quality control excluded loose/watery stools (Bristol types 6–7), exogenous-fluid-contaminated samples, or those with insufficient biomass (<200 mg). To prevent ex vivo microbial shifts, samples were kept at 4 °C, delivered to the lab within 30 min, and immediately aliquoted into sterile cryovials to avoid freeze-thaw degradation. Aliquots were flash-frozen in liquid nitrogen and stored at −80 °C until genomic DNA extraction, then shipped on dry ice (<−70 °C) to Novogene (Beijing, China) for sequencing.

The study was approved by the Institutional Review Board of Guangxi Medical University Cancer Hospital (No. KY2019009) and complied with the Declaration of Helsinki. All patients provided informed consent.

### 2.2 Fecal DNA extraction and 16S rRNA gene amplicon sequencing

Bacterial DNA was extracted from fecal samples using the cetyltrimethylammonium bromide method. The V3–V4 region of the 16S rRNA gene was amplified (341F/806R), purified, and sequenced (Illumina NovaSeq6000). Reads were processed to obtain and analyze Amplicon Sequence Variants (ASVs).

### 2.3 16S rRNA data analysis

Taxonomy was assigned using QIIME2 ‘s (Newsome et al., 2022) classify-sklearn with the Silva 138.1 database. Alpha and beta diversity (weighted/unweighted UniFrac- analysis of molecular variance [AMOVA]) and abundance analyses used normalized ASV data. Linear discriminant analysis effect size (LEfSe) identified differentially abundant biomarkers. PICRUSt2 predicted metabolic pathways (Douglas et al., 2020).

### 2.4 ASV biomarker identification and model construction, validation

We normalized the 16S ASV data and handled missing values using Multiple Imputation (MI) via the rice R package (v3.16.0). MI generated ten cross-validated datasets to enhance model stability and reduce overfitting (Huang et al., 2024). Subsequently, three robust feature selection methods—Boruta (Guindo et al., 2021), LASSO regression (Poudel et al., 2023), and the Random Forest (RF) algorithm (Ren et al., 2019; Wu et al., 2024) —were employed to identify stable ASVs. The R packages glmnet (v4.1-4), Boruta (v8.0.0), and RandomForest (v4.7-1.2) were used to implement the algorithm. Features were retained only if selected by at least two algorithms.

For model construction and training evaluation, we used a 10-fold cross-validation based on ASV expression profiles from the training set. The Synthetic Minority Over-sampling Technique (SMOTE) (Shi et al., 2022) algorithm was employed to handle sample distribution imbalances during training. We built GBM, RF, and XGBoost models (Hyperparameters for all machine learning models were systematically optimized via grid search with cross-validation, with the final optimal configurations summarized in **Supplementary Table 1**), using receiver operating characteristic (ROC) curves to assess the performance of the biomarkers and the models’ effectiveness. The SHapley Additive exPlanations (SHAP) (Hertel et al., 2026) method was used to explain the prediction process of the model. Chongqing regional cohort data were used as an independent external validation set. Model performance was evaluated using a comprehensive suite of metrics, including sensitivity, specificity, positive predictive value (PPV), negative predictive value (NPV), accuracy, the standard F1-score (F1-positive), and the negative F1-score (F1-negative). To evaluate the models’ performance as a clinical exclusionary tool for risk stratification, the F1-negative was employed as a derivative of the standard F1-score to specifically measure the reliability of identifying low-risk candidates. It was calculated as the harmonic mean of specificity and NPV:

F1-negative = 2 *(Specificity*NPV)/(Specificity + NPV).

Decision curve analysis (DCA) quantified clinical utility by assessing the net benefit of accurately identifying non-PHLF patients, thereby quantifying exclusionary value.

### 2.5 Statistical analysis

The demographic and clinical characteristics before surgery were compared between the PHLF and non-PHLF groups using R software (version 4.5.1). Statistical significance was defined as a two-sided p-value < 0.05. QIIME2 (Newsome et al., 2022) was used to evaluate α-diversity, whereas β-diversity was analyzed using non-metric multidimensional scaling (NMDS) and principal coordinate analysis (PCoA) with R software (version 4.0.3) and the ‘PCoA,’ ‘stats,’ and ‘ggplot2’ packages. A linear discriminant analysis (LDA) score threshold of three was employed using LEfSe software. The Wilcoxon rank-sum test identified microflora differences. PICRUSt2 pathway prediction relies on the t-test, we have analyzed the data applying the Benjamini-Hochberg False Discovery Rate (FDR). Spearman correlation analysis was then employed to assess the associations between the differential GM and predicted functional pathways. While the ROC effect was demonstrated by the area under the curve (AUC). The modeling analysis was performed in the R statistical environment (version 4.4.1). Missing values were imputed via mice (v3.16.0). Feature selection was conducted sequentially using Boruta (v8.0.0) and LASSO regression via glmnet (v4.1-7). Multivariate logistic regression analysis was performed to evaluate whether preoperative altered gut microbiota features (with relative abundance analyzed per 1% increase) served as independent predictors for PHLF, with prealbumin (PA) and INR adjusted as covariates to control for baseline clinical confounders. Machine learning models were constructed using RandomForest (v4.6-14) and the caret framework (v7.0-1). Model performance was evaluated using yardstick (v1.3.1), and model interpretability was visualized via KernelSHAP using kernelshap (v0.7.0).

## 3 Results

### 3.1 Baseline clinical characteristics

**Supplementary Table 2** provides an overview of the clinical symptoms and demographic details of the training set. Prealbumin levels and international normalized ratio levels differed significantly between the bo.PHLF and nbo.PHLF groups. None of the indices in the internal or independent external validation sets showed significant differences (**Supplementary Tables 10**, **11**).

### 3.2 α- and β-diversity

**Figure 1:**
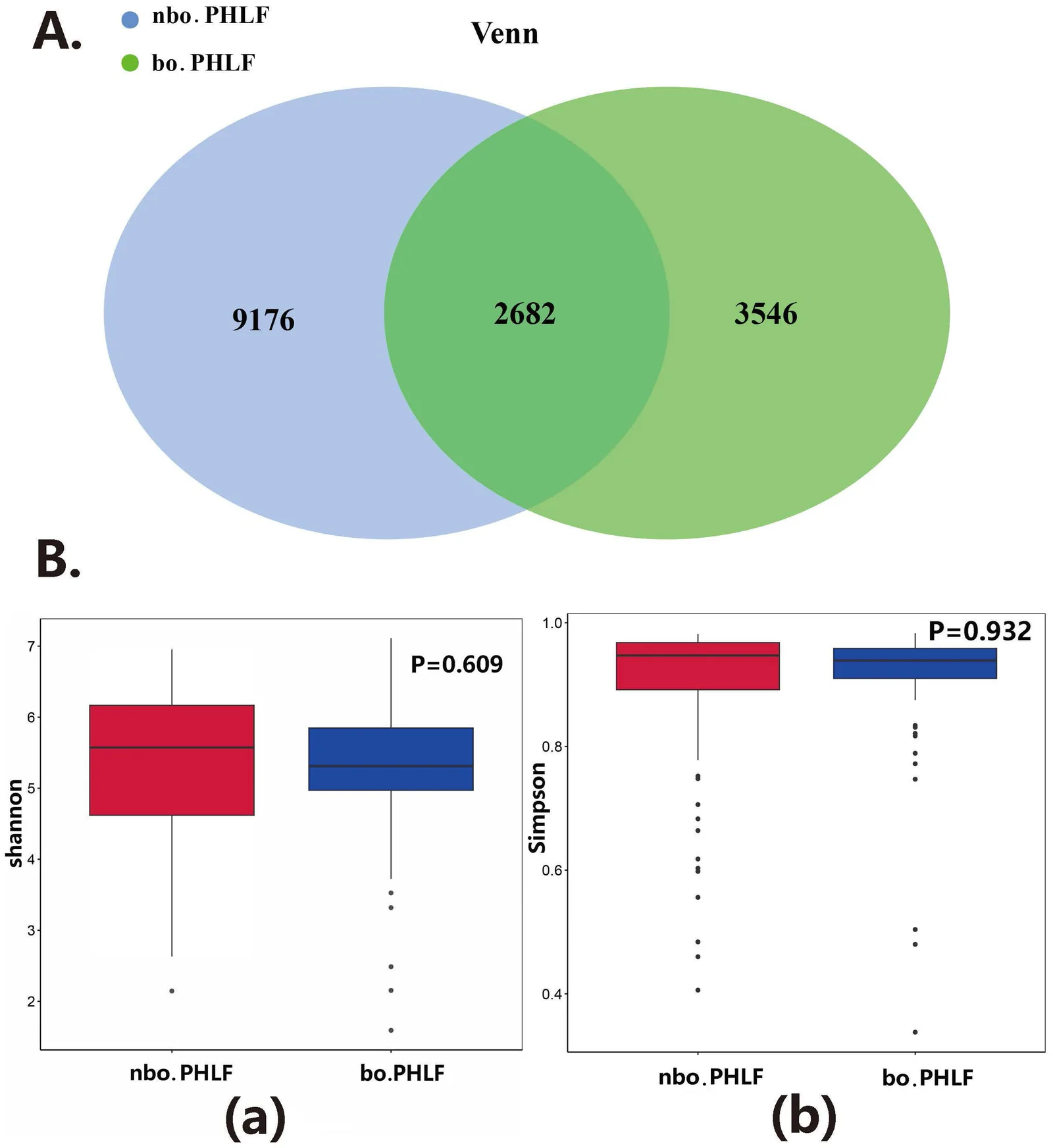
Identification of gut microbe using 16S rRNA analysis. **(A)** Venn diagrams show the common ASVs among bo.PHLF Group and nbo.PHLF Group. **(B)** bo.PHLF Group and nbo.PHLF Group comparison of α-diversity using shannon and simpson. *0.01<p ≤ 0.05. ASVs, Amplicon Sequence Variant; bo.PHLF, post-hepatectomy liver failure before hepatectomy; nbo.PHLF, post-hepatectomy without liver failure before hepatectomy.

**Figure 2:**
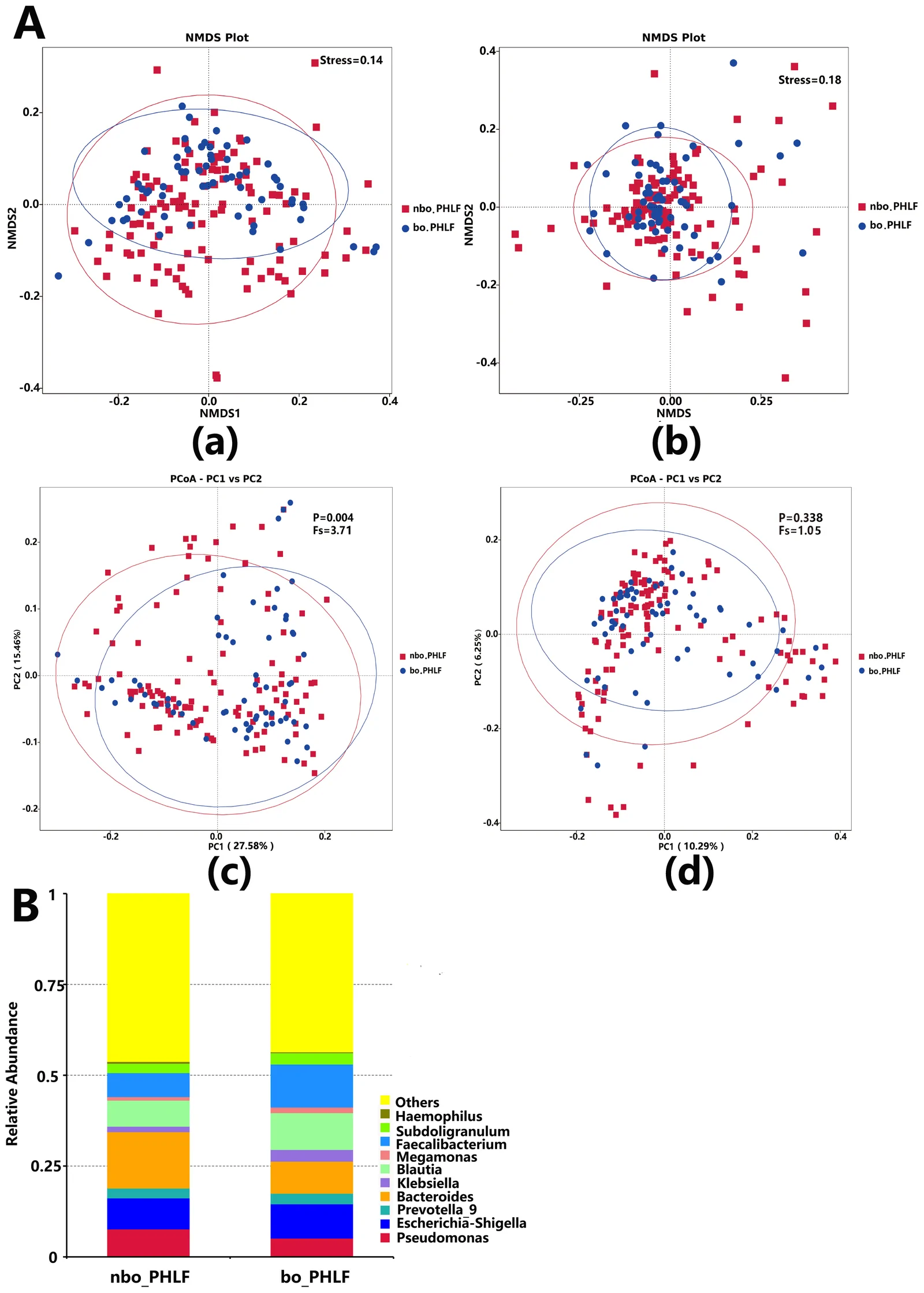
β-diversity analysis and relative abundance of the top 10 gut microbiome among groups bo.PHLF and nbo.PHLF. **(A)** β-diversity analysis. (a) NMDS plot based on weighted UniFrac distances b)NMDS plot based on unweighted UniFrac distances. (c) PCoA plot based on weighted UniFrac distances, (d) PCoA based on unweighted UniFrac distances. NMDS, non-metric multidimensional scaling; PCoA, primary coordinate analysis; stress values <0.2 were considered good after dimensionality reduction. p < 0.05 is statistically significant. **(B)** Relative abundance of the top 10 gut microbiome among groups bo.PHLF and nbo.PHLF at genus level. bo.PHLF, post-hepatectomy liver failure before hepatectomy; nbo.PHLF, post-hepatectomy without liver failure before hepatectomy.

ASVs represent different microbial taxa. In total, 2682 ASVs were found to be shared between the bo.PHLF and nbo.PHLF groups in the training set (**Figure 1A**). Furthermore, the Shannon (P = 0.609) and Simpson indices (P = 0.932) (**Figure 1B**) as well as the Chao1 (P = 0.157) and googe_coverage indices (P = 0.159) (**Supplementary Figure 1**) revealed no notable differences between the groups, suggesting comparable α-diversity. β-diversity for NMDS and PCoA was assessed using both weighted and unweighted UniFrac distances (**Figure 2A**). The NMDS analysis revealed notable differences in microbiome composition and abundance between the bo.PHLF and nbo.PHLF groups, with weighted UniFrac distance stress at 0.14 and unweighted at 0.18. PCoA and AMOVA tests using weighted UniFrac confirmed these differences (p = 0.004), whereas the unweighted UniFrac did not show significant differences (p = 0.338). The GM composition is different in patients with bo.PHLF compared to those with nbo.PHLF, as indicated by our results.

### 3.3 Specific GM signature in bo.PHLF and nbo.PHLF patients before surgery

In the training set, we assessed the ten core taxa with the highest abundance at various taxonomic levels, including phylum, class, order, family, genus, and species, between the bo.PHLF and nbo.PHLF groups (**Figure 2B**; **Supplementary Figure 2**), with a primary focus on the genus level to ensure taxonomic accuracy.

**Figure 3:**
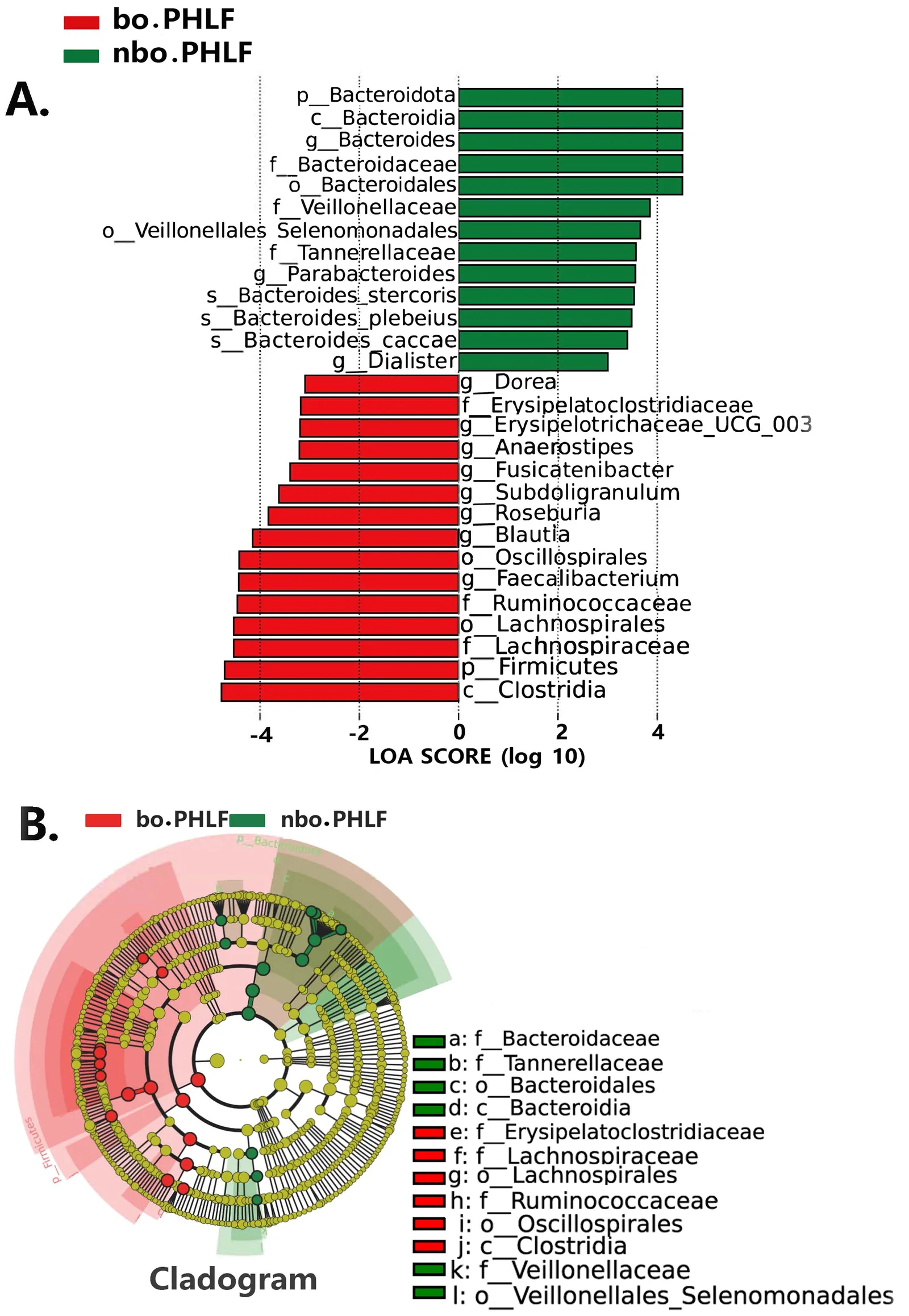
Bacterial taxa that best characterize the groups were identified by using LDA of effect size (LEfSe) on ASV tables among bo.PHLF Group vs. nbo.PHLF Group. **(A)** The bar plot based on the LDA selection. **(B)** Cladogram representing the taxonomic hierarchical structure. The corresponding precise LDA scores and P values for all 11 differential genera are detailed in **Supplementary Table 3**. LDA, linear discriminant analysis; ASVs, Amplicon Sequence Variant; bo.PHLF, post-hepatectomy liver failure before hepatectomy; nbo.PHLF, post-hepatectomy without liver failure before hepatectomy.

The most noteworthy genera identified in the training cohort exhibited distinct enrichment profiles between the two groups. Specifically, the mean relative abundances in the bo.PHLF versus nbo.PHLF groups were as follows: *Pseudomonas* (5.14% vs. 7.66%), *Escherichia-Shigella* (9.43% vs.8.53%), *Prevotella*_9 (2.91% vs. 2.72%), *Bacteroides* (8.82% vs. 15.52%), *Klebsiella* (3.23% vs. 1.50%), *Blautia* (10.13% vs. 7.18%), *Megamonas* (1.54% vs. 0.96%), *Faecalibacterium* (11.85% vs. 6.60%), *Subdoligranulum* (3.11% vs. 2.58%), and *Haemophilus* (0.25% vs. 0.51%). LEfSe revealed distinct GM. bo.PHLF was enriched in eight genera (e.g., *Dorea*, *Erysipelotrichaceae_UCG_003*, *Anaerostipes*, *Fusicatenibacter*, *Subdoligranulum*, *Roseburia*, *Blautia*, and *Faecalibacterium*), while nbo.PHLF was augmented with *Bacteroides*, *Parabacteroides*, and *Dialister* (**Figure 3**; **Supplementary Table 3**).

### 3.4 GM functional signatures

**Figure 4:**
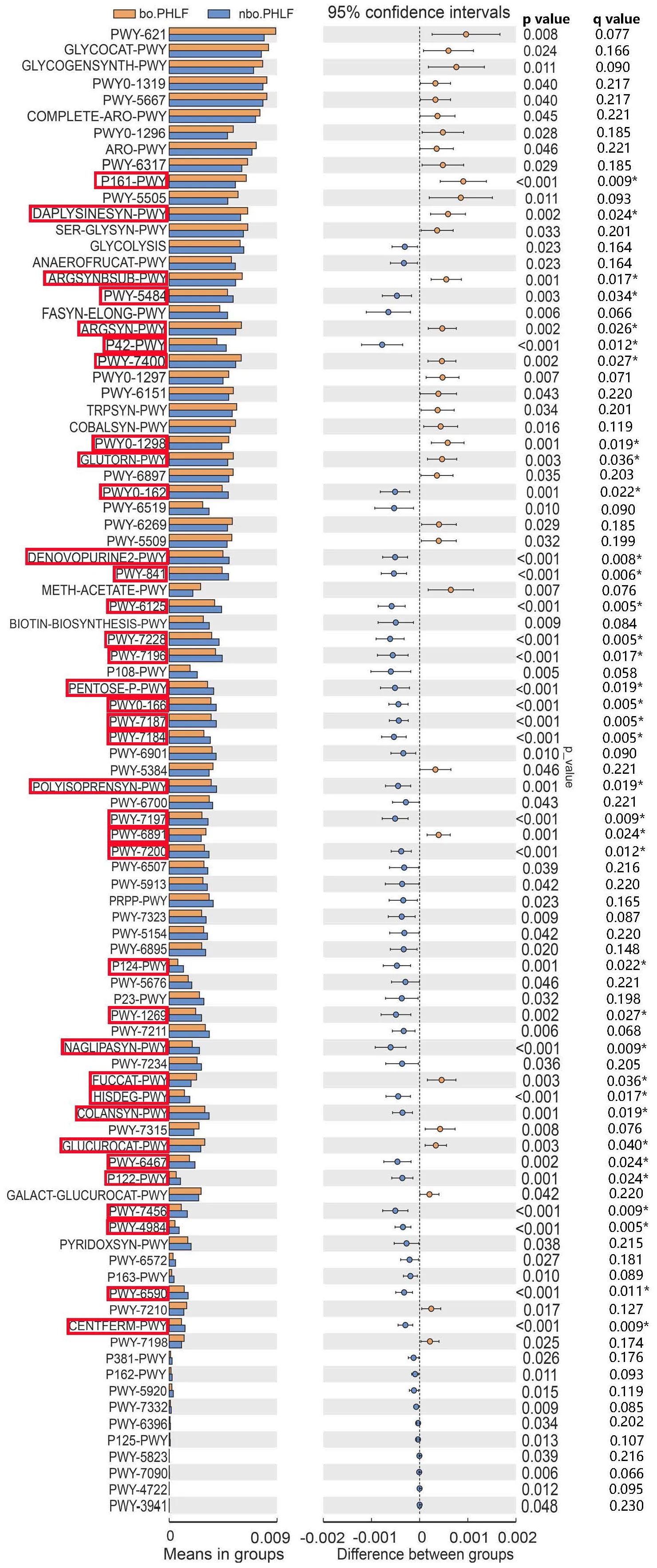
PICRUST2 prediction of functional alteration caused by gut microbiota change in bo.PHLF vs. nbo.PHLF. bo.PHLF, post-hepatectomy liver failure before hepatectomy; nbo.PHLF, post-hepatectomy without liver failure before hepatectomy; Statistically significant (q value)*; The red box highlights the pathway subsequent to FDR correction.

**Figure 5:**
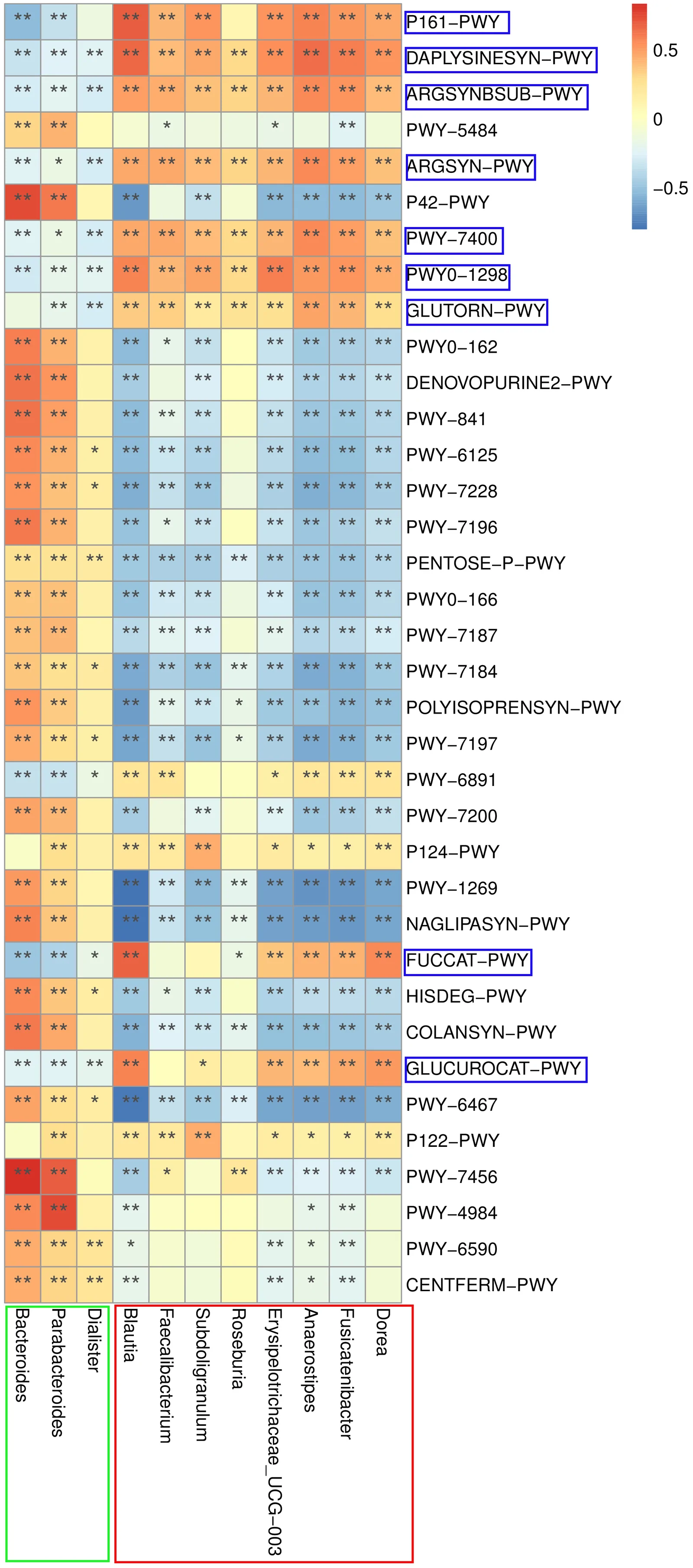
Correlation heatmap between key gut microbiota and functional metabolic pathways. The horizontal axis represents the microbial genera, categorized into the nbo.PHLF group (enclosed in the green box) and the bo.PHLF group (enclosed in the red box). The vertical axis displays the functional metabolic pathways, where pathways enclosed in blue boxes signify upregulated pathways, and those without boxes denote downregulated pathways. The color scale on the right indicates the correlation coefficient, ranging from blue (negative correlation)/-0.5/to red (positive correlation)/0.5/. Asterisks indicate statistical significance (*p < 0.05, **p < 0.01).

To elucidate the functional shifts in the gut microbiota, PICRUSt2-based functional prediction analysis was conducted to compare the bo.PHLF and nbo.PHLF groups. Following FDR-corrected multiple testing, the initial 91 differentially abundant pathways were rigorously refined to 36 highly significant functional alterations, comprising 10 upregulated and 26 downregulated pathways in the bo.PHLF group (**Supplementary Table 4**; **Figure 4**). Overall, functional profiling demonstrated a distinct redirection of microbial metabolic flux in the bo.PHLF group. Specifically, the microbiota associated with bo.PHLF exhibited significant enrichment in both nucleoside catabolic processes and specific biosynthetic pathways for basic and essential amino acids, including lysine, ornithine, and diverse arginine biosynthetic pathways; correlation analysis confirmed (**Figure 5**) that these functional modules were all robustly and positively correlated with bo.PHLF-specific taxa. Conversely, the bo.PHLF group exhibited profound, system-wide suppression of central carbon metabolism and energy-generating fermentation processes. Critical energy-producing pathways—encompassing glycolysis, the truncated TCA cycle, and the pentose phosphate pathway (PPP) and SCFA-producing butyrate metabolism—were markedly downregulated. Additionally, nucleotide homeostasis was disrupted via the synchronized downregulation of *de novo* and salvage pathways for purine and pyrimidine biosynthesis, including pyrimidine ribonucleotides, purine nucleotides, guanosine nucleotide synthesis, and pyrimidine ribonucleoside/deoxyribonucleoside metabolism. Structural biosynthetic pathways essential for bacterial cell envelope integrity and the lipopolysaccharide (LPS) matrix (primarily lipid biosynthesis), alongside histidine degradation and the urea cycle, were also significantly diminished. Heatmap analysis revealed that the signature taxa of the bo.PHLF group were prominently and negatively correlated with these suppressed metabolic pathways, whereas the nbo.PHLF-associated microbiota exhibited the exact inverse pattern. Collectively, these findings suggest that the progression of PHLF may be associated with preoperative metabolic alterations in the GM.

### 3.5 Establishment and verification of a prediction model for GM as a characteristic biomarker

**Figure 6:**
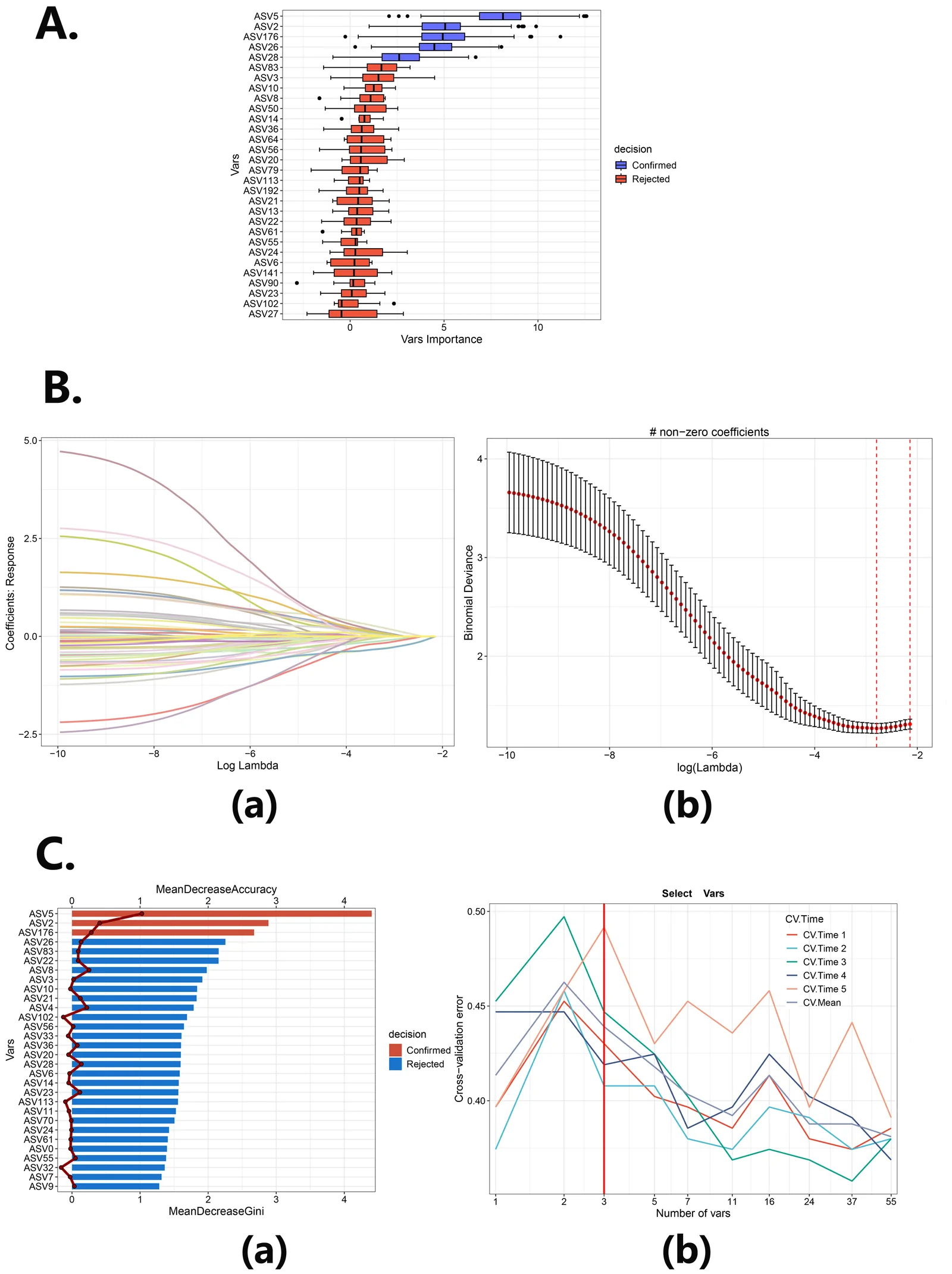
Screening Characteristics ASVs. **(A)** Boruta algorithm; **(B)** LASSO regression; **(C)** Random forest feature screening: (a) Bar chart shows the importance of Top30ASVs, (b) The line chart shows the process of determining the optimal feature number of features. ASVs, Amplicon Sequence Variant.

**Figure 7:**
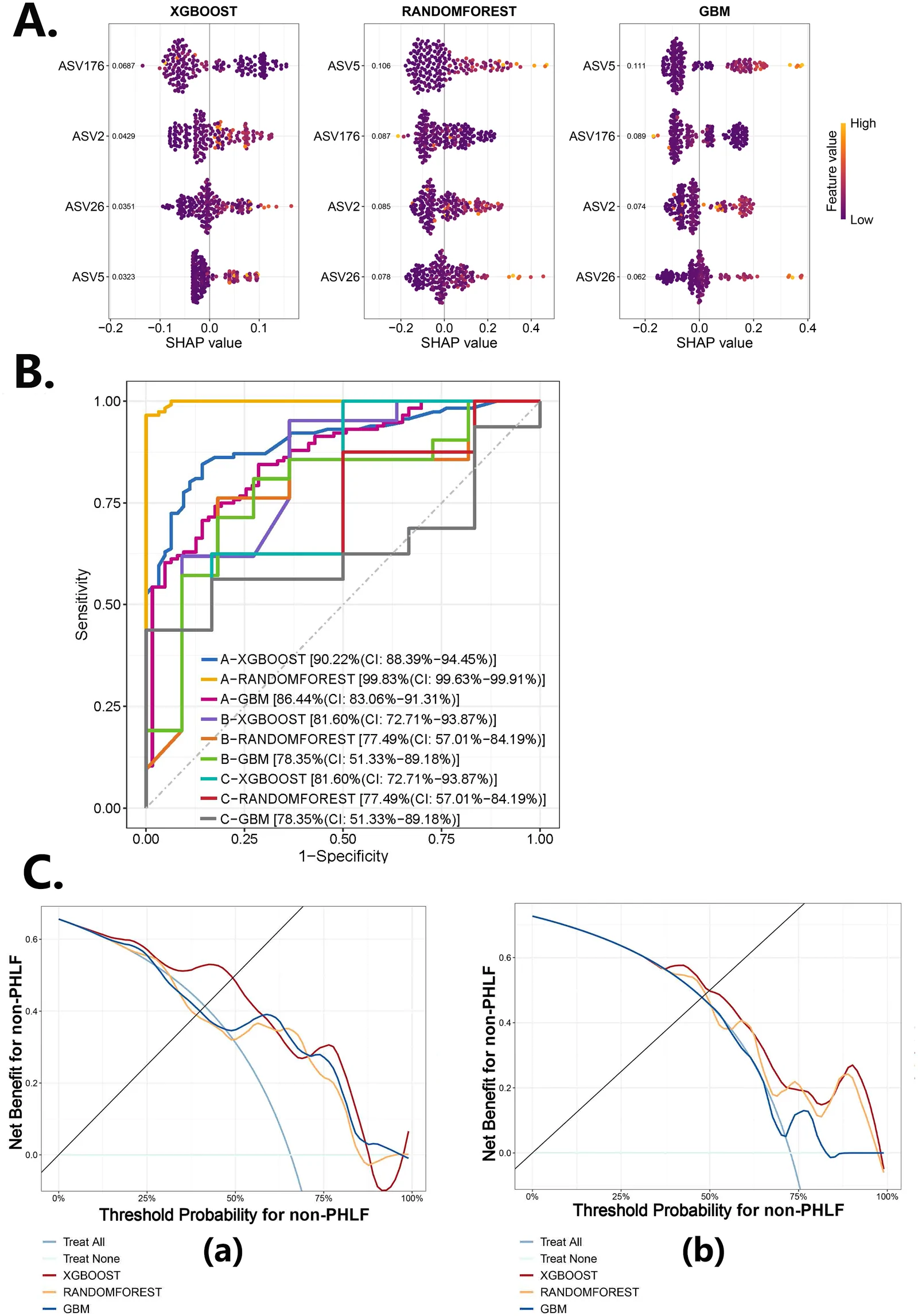
Machine learning model construction, performance evaluation, and clinical utility analysis based on the 4-ASV microbial signature. **(A)** SHAP (SHapley Additive exPlanations) summary plots for model interpretability. ASVs: Amplicon Sequence Variant; **(B)** Receiver operating characteristic (ROC) curves evaluating model efficacy. The multi-line plot controls and compares the predictive and exclusionary performance of the three machine learning frameworks across different validation levels [indicated as cohorts **(A–C)**]. The area under the curve (AUC) percentages alongside their respective 95% confidence intervals (CIs) are specified in the bounding index.; **(A)** group:The training set; **(B)** group: The internal validation set; **(C)** group:The independent external validation Set. **(C)** Decision curve analysis (DCA) for clinical net benefit. The decision curves quantify the clinical utility of the machine learning algorithms by plotting the threshold probability against the net benefit of accurately identifying non-PHLF patients (rule-out clinical value). (a) Internal Validation set, (b) Independent External Validation Set. The reference baselines “Treat All” (light blue line) and “Treat None” (grey line) are used to benchmark the net benefit curves of the XGBoost (red), RANDOMFOREST (orange), and GBM (dark blue) configurations. X-axis:Threshold probability for non-PHLF, Y-axis: Net clinical benefit for identifying patients non-PHLF; non-PHLF; post-hepatectomy without liver failure.

We designed a strategy based on the reference GM biomarkers to establish an early clinical prediction model for PHLF using sequencing data. Initially, **Supplementary Table 5** illustrates the characteristic reference ASVs we identified for the bo.PHLF (*n* = 63) and nbo.PHLF (*n* = 116) groups. Applying preprocessed data in conjunction with Boruta (**Figure 6A**), LASSO regression method (**Figure 6B**), and RF feature screening method (**Figure 6C**), we calculated the union and obtained four characteristic ASVs. The SHAP bee colony plot (**Figure 7A**) was utilized to demonstrate the strong correlation between the chosen ASV features and the predicted outcomes. ASV176 stood out as a predominant negative determinant, with its elevated abundance exhibiting a clear clustering with negative SHAP values. In marked contrast, ASV5, ASV26, and ASV2 served as positive determinants, with their heightened abundance profiles consistently mirroring positive SHAP distributions. To clarify the independent role of these four signature ASVs in PHLF risk, we performed multivariate logistic regression adjusted for baseline confounders (PA and INR), treating microbial abundance as a continuous variable per 1% increment (**Supplementary Table 6**). Within the individually adjusted models (Model A), the elevated abundances of ASV2, ASV5, and ASV26 were substantiated as independent risk factors for PHLF. Intriguingly, when all clinical and microbial covariates were integrated into the full model (Model B), only ASV5 and ASV26 maintained robust independent predictive power. Although statistical significance in the fully adjusted model was confined to ASV5 and ASV26 (P < 0.05), we retained the complete 4-ASV panel to capture the non-linear, synergistic microecological interactions at play. Subsequently, 10-fold cross-validation was employed for performance evaluation, and the GBM, RF, and XGBoost models were trained to establish prediction models for PHLF status, conducting both internal verification and independent verification. Model performance was systematically assessed across the training, internal validation, and external validation cohorts, leveraging a comprehensive set of metrics including accuracy, PPV, NPV, F1-positive, F1-negative, and AUC (**Figure 7B**; **Supplementary Tables 7**–**9**). In the training cohort, the RF model attained the highest AUC of 99.83%, followed by the XGBoost model at 90.22% and the GBM model at 86.44%. The classifiers retained robust discrimination capability in the internal validation cohort, with AUC values of 81.60% for XGBoost, 78.35% for GBM, and 77.49% for RF. However, in the independent external validation cohort from Chongqing, a marked divergence emerged in class-specific metrics. Driven by inherent geographic and dietary heterogeneity between Guangxi and Chongqing cohorts, the F1-positive (targeting PHLF identification) showed expected decline, predominantly due to reduced positive sensitivity.

Notably, when assessed under the preoperative “rule-out” mode, the models demonstrated potent exclusionary capacity. Both XGBoost and RF achieved 81.25% Specificity, with GBM reaching 72.22%, while all three models maintained a consistent NPV of 81.25%. Critically, the F1-negative—reflecting the model’s reliability in accurately identifying low-risk candidates—remained exceptionally stable: 81.25% for XGBoost, 81.25% for RF, and 76.47% for GBM in the external validation cohort. Concurrently, overall discriminative capacity varied across the algorithms, with the XGBoost model demonstrating the highest diagnostic efficacy (AUC = 78.12%), followed closely by the RF model (AUC = 71.88%). As demonstrated by the DCA, our 4-ASV microbial model exhibits profound clinical utility for preoperative risk stratification targeting non-PHLF status. In the internal validation set (**Figure 7C**-a), all three models outperformed default strategies within a wide threshold range of 25%-85%. In the independent external validation set (**Figure 7C**-b), this effective exclusionary window was successfully confirmed across a wider threshold range of 35% to 98%. Significantly, the default ‘Treat All’ strategy ceased to be beneficial at about 73%, whereas the XGBoost and RF models sustained a robust net benefit rebound within the high-threshold spectrum (75%–98%), peaking at 0.26 and 0.24, respectively, at about a 90% threshold.

## 4 Discussion

Given the high incidence of liver cancer in China, PHLF poses a formidable challenge to both clinicians and patients. The onset of PHLF is strongly associated with poor prognosis in HCC (Bartholomä et al., 2025), underscoring the critical necessity of rigorous perioperative management and preoperative intervention. Although accumulating evidence links the GM to various hepatic pathologies, the specific GM alterations distinguishing PHLF from non-PHLF patients, and their clinical implications—particularly regarding preoperative PHLF risk prediction—remain largely uncharted. In this study, we characterized the GM profiles of the bo.PHLF and nbo.PHLF cohorts and identified four robust ASV-level microbial markers. Subsequently, multicenter machine-learning models were developed and rigorously validated. Remarkably, to the best of our knowledge, this work constitutes one of the pioneering demonstrations that ASV-level markers can offer a highly specific strategy with exceptional diagnostic rule-out performance for preoperative PHLF risk stratification. Its successful cross-regional validation highlights the model’s potential as a high-fidelity screening adjunct within multistage decision-support systems, offering considerable promise for advancing precision medicine and defensive strategies in perioperative management.

No significant difference in α-diversity of the intestinal microbiota was observed between HBV-HCC patients with and without PHLF, however, β-diversity differed significantly. This indicates GM dysbiosis despite comparable overall community structure. While NMDS analysis based on UniFrac distance effectively distinguished samples irrespective of abundance, incorporating both abundance and presence-absence data enhanced discriminatory power. The pronounced differences observed in Weighted UniFrac metrics emphasize significant shifts in the relative abundance of specific microbial phylogenetic lineages between the bo.PHLF and nbo.PHLF groups. The pre-existing heterogeneity in the GM prior to surgery may serve as a proxy for nuanced disparities within the host’s systemic milieu, underlying hepatic functional compromise, or metabolic fluctuations orchestrated through the gut–liver axis (Chen et al., 2020; Shi et al., 2026). Perturbations in gut microbial homeostasis, coupled with concomitant alterations in specific microbial taxa (Dong and Perdew, 2020), may have the capacity to exacerbate systemic inflammatory responses and modulate circulating metabolomic profiles. Such perturbations may, in turn, subtly undermine the liver’s functional reserve, thereby impairing its regenerative potential following hepatectomy. Accordingly, these distinctive preoperative microbial signatures establish a compelling biological foundation for the adoption of GM-based biomarkers as a non-invasive modality to facilitate preoperative risk stratification for PHLF. Focusing on the genus level optimizes analytical accuracy, as amplicon sequencing typically resolves taxonomic distinctions most effectively at this resolution (Grządziel and Gałązka, 2019). Our study revealed distinct shifts in 10 GM genera between HBV-HCC patients with and without PHLF (bo.PHLF vs. nbo.PHLF), LEFSe analysis further identified 11 differentially abundant genera between the two cohorts. In nbo.PHLF patients, *Bacteroides*, *Parabacteroides*, and *Dialister* were enriched, while bo.PHLF patients exhibited elevated levels of *Dorea*, *Erysipelotrichaceae_UCG_003*, *Anaerostipes*, *Fusicatenibacter*, *Subdoligranulum*, *Roseburia*, *Blautia*, and *Faecalibacterium*. Prior studies demonstrate that in chronic liver disease, *Bacteroides* and *Parabacteroides* are linked to favorable metabolic profiles, reinforcing intestinal barrier integrity and stabilizing the internal environment (Lv et al., 2022; Wang et al., 2022). Wang et al. reported that murine intestinal *Bacteroides* alleviated liver injury and preserved liver function by suppressing CD95-dependent hepatocyte apoptosis (Wang et al., 2022). Additionally, liver disease patients show reduced oral-derived *Dialister* abundance compared to healthy controls, suggesting its beneficial role and therapeutic potential for liver disorders (Li et al., 2020a). Sodium alginate-pectin (AP) microgels have been shown to mitigate acute liver injury by reducing pathogenic *Dorea*, thereby attenuating inflammation and restoring the intestinal barrier (Zhuge et al., 2020). Our observation of *Dorea* enrichment in bo.PHLF patients implies a potential association with aggravated liver injury. *Erysipelotrichaceae_UCG_003* is associated with aging and impairs the regenerative capacity and metabolism of multiple systems, functioning as a potential pathogen that drives unhealthy aging and may compromise liver regeneration and repair (Singh et al., 2019). *Anaerostipes* enrichment historically implies enhanced butyrate synthesis (Cui et al., 2025), however, butyrate has a concentration-dependent duality. While physiological butyrate maintains gut–liver barrier homeostasis (Pan et al., 2023), pathological overproduction under perioperative stress can paradoxically trigger hepatocyte cytotoxicity and expansion of immunosuppressive Treg cells. while (Behary et al., 2021). Thus, these butyrate-producing consortia defy blanket categorization as wholly beneficial or harmful. Persistent proinflammatory activation is critical for the progression from hepatitis to liver cancer (Muthukutty et al., 2026), and such activation may be more pronounced in the bo.PHLF group, potentially counteracting the immunomodulatory and hepatoprotective effects of *Fusicatenibacter* (Zhou et al., 2022). Moreover, chronic inflammation and poor metabolic control are linked to *Subdoligranulum* abundance (Chong et al., 2025). Autoimmune liver disease, a group of inflammatory hepatobiliary disorders characterized by immune dysfunction, is associated with significant *Roseburia* enrichment (Lou et al., 2020). Mucin-degrading bacteria (e.g., R-torque species) are elevated in established Crohn’s disease, where they promote the expansion of other mucin-utilizing bacteria, thereby damaging the intestinal epithelial barrier. *Blautia*, another mucin-degrader, is markedly increased in inflammatory contexts (Garay et al., 2023). Our previous work found that patients who developed post-extended hepatectomy liver failure had elevated preoperative *Faecalibacterium* levels; postoperatively, although its abundance declined significantly, it remained higher than in non-PHLF patients. This reduction may reflect *Faecalibacterium*’s resilience to liver failure and competitive constraints from other microbes (Peng et al., 2022). Collectively, these findings indicate that specific GM taxa play a pivotal role in shaping the bo.PHLF and nbo.PHLF status of HBV-HCC patients.

PICRUST2 analysis identified significant differences in microbiome functional profiles between the bo.PHLF and nbo.PHLF cohorts, particularly within core metabolic pathways. Critically, the convergent integration of the microbial ornithine/arginine biosynthesis pathway and the host hepatic urea cycle is essential for modulating systemic ammonia elimination and glutamate homeostasis, processes that are closely intertwined with acute liver injury and severe liver dysfunction (Thomsen et al., 2023). Under physiological circumstances, hepatic ammonia detoxification is fundamentally contingent upon precise metabolic zonation: periportal hepatocytes utilize glutamine to facilitate the biosynthesis of urea and glutamate, whereas perivenous hepatocytes scavenge residual ammonia via glutamine synthetase (GS). The downregulation of the urea cycle predicted in this study robustly indicates a profound derangement of this detoxification circuit, thereby potentially inducing the pathological accumulation of ammonia and glutamate (Ismaiel et al., 2026). This metabolic collapse inflicts a dual pathological insult upon the post-surgical remnant liver: notably, the accumulated glutamate at high concentrations exerts direct cytotoxic effects, thereby exacerbating hepatocellular injury and cell death (Santiago-Castañeda et al., 2022). Conversely, the blockade of both the urea cycle and the GS pathway constrains glutamine availability. Given that glutamine serves as an indispensable precursor for nucleotide biosynthesis (Tang et al., 2022), this metabolic defect aligns closely with the predicted phenotype characterized by “downregulated nucleotide synthesis coupled with accelerated degradation.” Such a deficit may deprive the liver of the essential substrates required for post-resection repair and compensatory regeneration. Moreover, glutamate functions as a lysine precursor and is integrated into the urea cycle (Li et al., 2023). Intriguingly, physiologically, the liver activates glycolytic flux (Yasaka et al., 2025) or deploys compensatory mitochondrial mechanisms, such as α-ketoglutarate reductive carboxylation (a manifestation of incomplete TCA cycle reduction), to intervene at upstream metabolic nodes driving glutathione and nucleotide synthesis, thereby upholding essential cellular survival (Shen et al., 2020). The predicted uniform downregulation of these two pathways’ functional activities in this study signifies that hepatocytes have surpassed a critical threshold, forfeiting their fundamental bioenergetic resilience. The decline in gut-derived butyrate may be linked to this metabolic crisis, potentially depriving the liver of exogenous energy substrates and pivotal signaling molecules indispensable for sustaining enterohepatic homeostasis (Kõiv et al., 2020). Even more detrimental is the fact that, given the PPP serves as the primary intracellular source of nicotinamide adenine dinucleotide phosphate (NADPH), its downregulation entails a complete collapse of the antioxidant defense machinery (Li et al., 2020b). Our observations revealed the simultaneous suppression of nucleotide and lipid biosynthetic pathways. This anabolic failure is mechanistically underpinned by two interrelated constraints: primarily, the obstruction of histidine degradation may disrupt the one-carbon folate metabolic pool (Pierce et al., 2019), hence eliminating the core carbon donor indispensable for purine and pyrimidine ring biosynthesis (Romoli et al., 2021). Second, given that lipid biosynthesis is an energy-intensive process heavily dependent on ATP and NADPH (Erlich et al., 2022), acute deficits of these metabolites inevitably drive a marked reduction in lipid biosynthesis. The concurrent deprivation of nucleotides and lipids obstructs DNA/RNA repair and cell membrane remodeling (Rogez et al., 2019; Pan et al., 2020). This thereby induces substantial impairment of liver cell proliferation and tissue regeneration. Taken together, while functional profiling via PICRUSt2 is inherently predictive, the expanding body of literature and bioinformatic evidence pointing to a dysregulated microbiome-host axis in PHLF are fully consistent with the correlations observed between metabolic pathways and cohort-specific taxa in this study. Consequently, future investigations incorporating metagenomics, targeted metabolomics, and *in vivo* validation are imperative to substantiate these predictive findings.

Recent evidence confirms that the GM serves as a distinctive biomarker for disease risk stratification. Leveraging GM as a predictive indicator, integrated with diverse machine-learning algorithms to construct predictive models, has been extensively adopted in clinical research. For instance, Caussy et al. established a gut microbiota-based random forest model for NAFLD-related cirrhosis, which delivered high specificity (91.6%) and an AUC of 0.87, offering a clinically feasible risk stratification tool (Caussy et al., 2019). Liu et al. developed an XGBoost model based on OTU profiles, achieving an AUC of 0.913 for prognostic risk stratification in urinary stone types (Liu et al., 2025). Wang et al. employed ASV-driven RF modeling to achieve an AUC of 0.796 in predicting preoperative lymph node metastasis in colorectal cancer patients—a core conclusion of this study. This model supports personalized, accurate non-invasive preoperative staging, offering a pivotal reference for advancing colorectal cancer clinical practice (Wu et al., 2025). In a Japanese cohort, GM was applied to stratify prostate cancer risk. Although sensitivity for identifying high-risk cases was limited, the final model attained an AUC of 0.81, preserving its utility in predictive stratification for treatment prognosis (Matsushita et al., 2021). In recent years, constrained by geographical disparities and dietary variations, the development of a cross-regional, universally applicable, and precisely stratified diagnostic model leveraging the intestinal microbiota continues to be beset by the profound predicament. We pinpointed four distinctive bacterial clusters and subsequently employed three distinct machine learning algorithms—namely XGBoost, Random Forest (RF), and Gradient Boosting Machine (GBM)—to construct the models. In our independent external validation cohort, these models demonstrated an expected decline in positive sensitivity (a reduced F1-positive score) but preserved robust specificity (81.25% for both XGBoost and RF, 77,22% for GBM), a high NPV, and an exceptionally stable F1-negative score exceeding 76%. This clinical performance profile precisely satisfies the criteria for a frontline preoperative “rule-out” tool, whose core mandate is to accurately identify low-risk candidates and preclude unnecessary conservative over-treatment. Crucially, the clinical utility of these strategies depend on evaluating the 4-ASV panel as a unified microecological index, rather than assessing individual taxa as independent linear predictors (Ren et al., 2019). This multivariate framework reconciles the fluctuating statistical significance observed across individual regression models. In the model refined by integrating individual signature bacteria with clinically pertinent metrics, ASV2, ASV5, and ASV26 were confirmed as indicators of dysbiosis. This pathogenic role is corroborated by SHAP profiling, which identified distinct positive risk weights for ASV2, ASV5, and ASV26, contrastingly paired with a negative homeostatic contribution from ASV176. When evaluated jointly alongside prealbumin—which consistently maintained independent predictive significance (P < 0.05) as a robust clinical anchor of liver reserve, ASV2 lost independent significance. This change occurs because ASV2 shares redundant, overlapping pathogenic variance with the more dominant ASV5 and ASV26 signals, acting as a co-linear marker of the same underlying bacterial cluster. Similarly, while the homeostatic marker ASV176 lacks global linear independence under strict baseline adjustment. Should the exclusion of ASV 2 or ASV 176 destabilize the microbial co-abundance network, it would consequently fall short of faithfully mirroring the entire microecological milieu characteristic of pathological conditions. By integrating synergistic microbial features, the joint 4-ASV model successfully captures baseline fluctuations along the gut-liver axis; as a highly specific screening modality, it provides biologically meaningful, independent diagnostic insights beyond those offered by conventional clinical parameters (Caussy et al., 2019). Undeniable, the distinct variations of AUC across the three machine learning models highlight their varying sensitivities to cross-regional microbiome heterogeneity. Specifically, the performance drop of the RF model—with its AUC plummeting from near-perfect discrimination in the training cohort to 71.88% in the external validation set — the discrepancy between the RF model’s high training AUCs and its validation fluctuations underscores the well-documented risk of overfitting on high-dimensional microbiome data. This cross-cohort performance degradation is largely driven by the intrinsic baseline ecological divergence between the two geographical populations. Although the GBM model exhibited a marginal attenuation in AUC during external validation, its NPV and F1- negative score retained remarkable strength. Intriguingly, despite the unexpectedly pronounced dietary divergence between Guangxi and Chongqing,

(Hou et al., 2020), external validation revealed that all three models exhibited strikingly robust and enduring exclusion accuracy. Indeed, the profound pathological stress, chronic inflammation, and metabolic remodeling intrinsic to advanced HBV-HCC appear to exert a uniform, dominant selective pressure on the gut ecosystem, effectively overriding regional environmental variations (Ren et al., 2019). However, this striking cross-algorithmic consistency demonstrates that the negative predictive elements within the 4-ASV signature are highly robust, overriding both mathematical differences and regional environmental variations. That these exclusionary metrics remained stable across distinct cohorts implies that the microecological homeostasis of the gut–liver axis driving perioperative resilience is deeply conserved in different HBV-HCC populations. toward Subsequently, DCA utilizing calibrated probabilities was implemented to quantify the potential clinical net benefit of this exclusionary approach in practical decision-making. All three models demonstrated a consistently superior standardized net benefit over default clinical strategies across an extensive threshold range extending up to 98% in the independent external cohort. Within the high-threshold domain, where clinical tolerance for diagnostic error is minimal, the sustained net benefit of XGBoost and RF near the 90% threshold underscores the models’ robust performance and potential for high-certainty risk exclusion.

From a clinical application perspective, the high negative predictive fidelity of this model holds value in precluding under-treatment stemming from overly cautious clinical decision-making. In major hepatectomies for HBV-HCC, balancing oncological radicality against future liver remnant volume remains a critical challenge. Overestimating PHLF risk in an inherently stable patient can prompt unwarranted clinical over-caution, such as denying curative-intent resection in favor of palliative care, or subjecting candidates to redundant portal vein embolization. Fortunately, the consistently stable and outstanding F1-negative scores across both internal and external validation cohorts robustly attest to these models’ clinical reliability as an exclusion instrument. By precisely stratifying low-risk surgical candidates preoperatively, these models safeguard eligible patients from unwarranted treatment constraints and facilitate their seamless integration into the Enhanced Recovery After Surgery pathway, thus refining perioperative resource allocation.

Furthermore, given that the GM not only reflects the systemic inflammatory and metabolic status of the gut-liver axis but also serves as a highly modifiable defensive system, our study highlights a promising prehabilitation avenue: perioperative microbiome-targeted interventions—such as tailored prebiotics or synbiotics—can be implemented to target and rectify the underlying dysbiosis in patients with borderline risk profiles. This proactive strategy empowers clinicians to remodel the microbial architecture of these borderline candidates into a resilient, low-risk homeostatic phenotype prior to surgical stress. Ultimately, integrating the 4-ASV signature as an early screening layer provides surgical teams with a reliable defensive benchmark. Complementing conventional clinical metrics, the GM model provides an independent microecological foundation that yields substantial incremental diagnostic value, effectively widening the safety threshold for surgical decision-making. Despite the promising insights yielded by this study, several limitations warrant acknowledgment. The relatively small sample size of the external validation cohort may, to some extent, constrain the generalizability of our findings. Owing to our highly stringent prospective inclusion criteria, the sample size accrued within the collection window was inevitably constrained. Nevertheless, this methodological rigor was paramount to eliminating confounding variables, preserving the integrity of biological signals, and yielding high-fidelity microbiome signatures. Although the 4-ASV model omits conventional perioperative parameters like ICG and resection volume, this standalone microbial signature statistically prevents overfitting and multicollinearity within a constrained validation space. This microbial signature captures independent biological variables, thereby precluding statistical redundancy with conventional laboratory metrics. As such, this panel serves to augment established prognostic tools, rather than supersede them. Consequently, future investigations will integrate metagenomic sequencing, targeted metabolomics, and animal model validation to unravel the precise mechanistic interplay between intestinal microecology and PHLF progression. Concurrently, we aim to expand the multi-center, large-scale cohort and conduct clinical trials of preoperative microbiome interventions. Furthermore, we will expedite the development of rapid detection kits for core microbial biomarkers.

In conclusion, we delineated systematic GM alterations in HBV- HCC patients with bo.PHLF and nbo.PHLF. Analyses at the ASV level establish the GM as a robust, noninvasive biomarker with high negative predictive performance for PHLF. Although the model’s direct sensitivity for predicting PHLF onset is relatively constrained, it demonstrates highly reliable rule-out capability in identifying non-PHLF candidates characterized by a homeostatic microbial ecosystem. This advantage facilitates optimal perioperative resource allocation and establishes a robust theoretical underpinning for the advancement of refined clinical prediction strategies and targeted perioperative microecological intervention protocols.

## Data Availability

The 16S rRNA amplicon sequencing data generated in this study have been deposited in the NCBI Sequence Read Archive (SRA) under the BioProject accession number PRJNA1475442.

## Supplementary material

Supplementary Figure 1 bo.PHLF Group and nbo.PHLF Group comparison of α-diversity using Chao1 and googs_coverage revealed. **(A, B)**. *P <0.05. ASV, Amplicon Sequence Variant; bo.PHLF, post-hepatectomy liver failure before hepatectomy; nbo.PHLF, post-hepatectomy without liver failure before hepatectomy.

Supplementary Figure 2 Relative abundance of the top 10 gut microbiome among groups bo.PHLF and nbo.PHLF. **(a)** phylum level, **(b)** class level, **(c)** order level, **(d)** family level, **(e)** species level. bo.PHLF, post-hepatectomy liver failure before hepatectomy; nbo.PHLF, post-hepatectomy without liver failure before hepatectomy.

## Glossary

AASLD: American Association for the Study of Liver Diseases
AFP: Alpha-fetoprotein
ALT: Alanine aminotransferase
AMOVA: Analysis of molecular variance
AST: Aspartate aminotransferase
ASV: Amplicon sequence variant
AUC/AUCs: Area(s) under the receiver operating characteristic curve
BCLC: Barcelona Clinic Liver Cancer
BMI: Body mass index
bo.PHLF: post-hepatectomy liver failure before hepatectomy
CT: Computed tomography
DCA: Decision curve analysis
EASL: European Association for the Study of the Liver
F1-negative: Negative F1-score
F1-positive: Standard F1-score
GBM: Gradient Boosting Machine
GM: Gut microbiota
GS: Glutamine synthetase
HBsAg: Hepatitis B surface antigen
HBV: Hepatitis B virus
HBV-HCC: Hepatitis B virus-related hepatocellular carcinoma
HCC: Hepatocellular carcinoma
HCV: Hepatitis C virus
ICG: Indocyanine green
INR: International normalized ratio
ISGLS: International Study Group of Liver Surgery
LASSO: Least absolute shrinkage and selection operator
LEfSe: Linear discriminant analysis effect size
LPS: Lipopolysaccharides
MI: Multiple Imputation
NADPH: Nicotinamide adenine dinucleotide phosphate
NAFLD: Non-alcoholic fatty liver disease
nbo.PHLF: post-hepatectomy without liver failure before hepatectomy
non-PHLF: post-hepatectomy without liver failure
NMDS: Non-metric multidimensional scaling
NPV: Negative predictive value
OTU: Operational taxonomic unit
PA: Prealbumin
PCoA: Principal coordinate analysis
PHLF: Post-hepatectomy liver failure
PPP: Pentose phosphate pathway
PPV: Positive predictive value
RF: Random Forest
SCFA/SCFAs: Short-chain fatty acid(s)
SHAP: SHapley Additive exPlanations
SMOTE: Synthetic Minority Over-sampling Technique
TCA: Tricarboxylic acid
