# Supplementary material for: Preoperative gut microbiota combined with machine learning for predicting post-hepatectomy liver failure in HBV-related hepatocellular carcinoma: a pilot study
Source: Front Cell Infect Microbiol. 2026 Aug 6;16:1877450. doi: 10.3389/fcimb.2026.1877450 (PMC13490075; doi:10.3389/fcimb.2026.1877450)
Supplement: Supplementary file 1 [file Table6.docx]

**Note Table S6A and Table S6B :** The input unit for each gut microbiota feature represents a 1% increase in relative abundance. Note: All numerical values are rounded to two decimal places, except for the OR , 95% CI and P-value which are presented with three decimal places to accurately demonstrate statistical significance without rounding the upper limit to 1.00. PA: Prealbumin ; INR: International Normalized Ratio

**Table S6A: Multivariate Logistic Regression Analysis of Individual Gut Microbiota Features Adjusted for Clinical Confounders (Model A)**

| Model / Variable | Coefficient (β) | Standard Error (SE) | OR (95% CI) | P-value |
| --- | --- | --- | --- | --- |
| Model 1 |  |  |  |  |
| PA | −0.01 | 0.003 | 0.992 (0.986–0.998) | 0.011 |
| INR | 2.24 | 1.684 | 9.352 (0.345–253.801) | 0.184 |
| ASV2 | 0.05 | 0.018 | 1.046 (1.010–1.084) | 0.013 |
| Model 2 |  |  |  |  |
| PA | −0.01 | 0.003 | 0.993 (0.987–0.999) | 0.023 |
| INR | 0.91 | 1.770 | 2.477 (0.077–79.486) | 0.608 |
| ASV5 | 0.07 | 0.021 | 1.076 (1.033–1.121) | <0.001 |
| Model 3 |  |  |  |  |
| PA | −0.01 | 0.003 | 0.992 (0.986–0.998) | 0.008 |
| INR | 2.64 | 1.729 | 13.949 (0.471–413.112) | 0.127 |
| ASV26 | 0.70 | 0.215 | 2.020 (1.327–3.076) | 0.001 |
| Model 4 |  |  |  |  |
| PA | −0.01 | 0.003 | 0.994 (0.988–0.999) | 0.037 |
| INR | 2.26 | 1.661 | 9.548 (0.368–247.899) | 0.175 |
| ASV176 | −0.60 | 0.913 | 0.551 (0.092–3.294) | 0.513 |

**Table S6B: Full Multivariate Logistic Regression Model with Simultaneous Adjustment (Model B)**

*Note: All clinical baseline confounders and the four gut microbiota features are integrated simultaneously into a single full model.*

| Variable | Coefficient (β) | Standard Error (SE) | OR (95% CI) | P-value |
| --- | --- | --- | --- | --- |
| Intercept | −0.86 | 2.122 | 0.425 (—) | 0.687 |
| PA | −0.01 | 0.003 | 0.990 (0.984–0.997) | 0.004 |
| INR | 0.88 | 1.885 | 2.412 (0.060–96.987) | 0.641 |
| ASV2 (per 1% increase) | 0.03 | 0.021 | 1.0301(0.989–1.074) | 0.146 |
| ASV5 (per 1% increase) | 0.08 | 0.022 | 1.082 (1.036–1.129) | <0.001 |
| ASV26 (per 1% increase) | 0.56 | 0.220 | 1.752 (1.138–2.697) | 0.011 |
| ASV176 (per 1% increase) | −0.43 | 0.9206 | 0.650 (0.107–3.949) | 0.640 |
