# Supplementary material for: Preoperative gut microbiota combined with machine learning for predicting post-hepatectomy liver failure in HBV-related hepatocellular carcinoma: a pilot study
Source: Front Cell Infect Microbiol. 2026 Aug 6;16:1877450. doi: 10.3389/fcimb.2026.1877450 (PMC13490075; doi:10.3389/fcimb.2026.1877450)
Supplement: Supplementary file 2 [file Table1.doc]

Table S1. Optimal hyperparameter configurations for the machine learning models.

| Model | Hyperparameter | Optimal Value |
| --- | --- | --- |
| XGBoost | nrounds/max_depth/eta/gamma | 3 / 2 / 0.2 / 0.25 |
|  | colsample_bytree/subsample/min_child_weight | 0.8 / 0.8 / 1 |
| Random Forest | mtry / ntree / nodesize | 2 / 240 / 2 |
| GBM | n.trees/interaction.depth/shrinkage/  n.minobsinnode | 200 / 5 / 0.001 / 3 |
