# Supplementary material for: Preoperative gut microbiota combined with machine learning for predicting post-hepatectomy liver failure in HBV-related hepatocellular carcinoma: a pilot study
Source: Front Cell Infect Microbiol. 2026 Aug 6;16:1877450. doi: 10.3389/fcimb.2026.1877450 (PMC13490075; doi:10.3389/fcimb.2026.1877450)
Supplement: Supplementary file 5 [file Table2.doc]

Table S2 Comparison of Clinical Characteristics Baseline between Training set before Surgery in 179 HBV-HCC Patients at Guangxi

| **Characteristics** | **bo.PHLF(n=63)** | **nbo.PHLF(n=116)** | **P value** |
| --- | --- | --- | --- |
| Age(year) |  |  | 0.611 |
| <45 | 27 (42.90) | 43 (37.70) |  |
| ≥45 | 36 (57.10) | 73 (62.30) |  |
| Gender(Sex) |  |  | 0.981 |
| Male | 54 (85.70) | 101 (87.10) |  |
| Female | 9 (14.30) | 15 (12.90) |  |
| BMI stage |  |  | 0.411 |
| 1 | 3 (4.80) | 8 (6.90) |  |
| 2 | 41 (65.10) | 83 (71.60) |  |
| 3 | 19 (30.20) | 25 (21.60) |  |
| smoking |  |  | 0.693 |
| No | 38 (60.30) | 65 (56.00) |  |
| Yes | 25 (39.70) | 51 (44.00) |  |
| drinking |  |  | 0.622 |
| No | 48 (76.20) | 83 (71.60) |  |
| Yes | 15 (23.80) | 33 (28.40) |  |
| child-pugh grade |  |  | 0.424 |
| A | 57 (90.50) | 110 (94.80) |  |
| B | 6 (9.50) | 6 (5.20) |  |
| TBIL(umol/L) |  |  | 0.277 |
| ≤17.1 | 46 (73.00) | 74 (63.80) |  |
| ＞17.1 | 17 (27.00) | 42 (36.20) |  |
| ALB(g/L) |  |  | 0.478 |
| ＜35 | 12 (19.00) | 16 (13.80) |  |
| ≥35 | 51 (81.00) | 100 (86.20) |  |
| ALT(U/L) |  |  | >0.999 |
| ≤40 | 44 (69.80) | 82 (70.70) |  |
| ＞40 | 19 (30.20) | 34 (29.30) |  |
| AST(U/L) |  |  | 0.726 |
| ≤40 | 29 (46.00) | 58 (50.00) |  |
| ＞40 | 34 (54.00) | 58 (50.00) |  |
| HBV-DNA(/mL) |  |  | 0.664 |
| ≤10^3^ | 20 (31.70) | 42 (36.20) |  |
| ＞10^3^ | 43 (68.30) | 74 (63.80) |  |
|  |  |  |  |
|  |  |  |  |
| Table S2 (Continue)Comparison of Clinical Characteristics Baseline between Training set before Surgery in 179 HBV-HCC Patients | | | |
| **Characteristics** | **bo.PHLF(n=63)** | **nbo.PHLF(n=116)** | **P value** |
| PT(s)  ≤13  ＞13  AFP(ng/mL)  ≤400  ＞400 | 42 (66.70)  21 (33.30)    34 (54.00)  29 (46.00) | 78 (67.20)  38 (32.80)    64 (55.20)  52 (44.80) | >0.999    >0.999 |
| Ascites |  |  | 0.664 |
| No | 58 (92.10) | 103 (88.80) |  |
| Yes | 5 (7.90) | 13 (11.20) |  |
| Portal hypertension |  |  | >0.999 |
| No | 55 (87.30) | 14 (12.30) |  |
| Yes | 8 (12.70) | 14 (12.30) |  |
| tumor size stage(cm) |  |  | 0.567 |
| ≤5 | 23 (36.50) | 48 (41.40) |  |
| ＞5, ≤10 | 22 (34.90) | 43 (37.10) |  |
| ＞10 | 18 (28.60) | 25 (21.60) |  |
| Liver resection involves more than |  |  | >0.999 |
| 3 segments |  |  |  |
| No | 42 (66.70) | 77 (66.40) |  |
| Yes | 21 (33.30) | 39 (33.60) |  |
| Laparoscopy or Open abdomen |  |  | 0.529 |
| Laparoscopy | 17 (27.00) | 38 (32.80) |  |
| Open abdomen | 46 (73.00) | 78 (67.20) |  |
| BCLC stage |  |  | 0.232 |
| 0 | 0 (0.00) | 6 (5.20) |  |
| A | 44 (69.80) | 69 (59.50) |  |
| B | 9 (14.30) | 20 (17.20) |  |
| C | 10 (15.90) | 21 (18.10) |  |
| CT Cirrhotic nodule  and Pathological  diagnosis |  |  | 0.461 |
| No | 32 (50.80) | 67 (57.80) |  |
| Yes | 31 (49.20) | 49 (42.20) |  |

Table S2 (Continue)Comparison of Clinical Characteristics Baseline between Training set before Surgery in 179 HBV-HCC Patients

| **Characteristics** | **bo.PHLF(n=63)** | **nbo.PHLF(n=116)** | **P value** |
| --- | --- | --- | --- |
| ICG excretion test |  |  | 0.069 |
| ≤10% | 54 (85.70) | 110 (94.80) |  |
| ＞10% | 9 (14.30) | 6 (5.20) |  |
| TBA(μmol/L) | 9.00(4.90-14.90) | 8.05(5.00-14.12) | 0.830 |
| PLT(10^9^/L) | 202.00(148.00-230.50) | 199.50(158.25-267.25) | 0.207 |
| HGB(g/L) | 144.00(131.00-153.00) | 143.00(131.75-152.25) | 0.965 |
| PA(mg/L) | 192.80(159.00-236.05) | 212.00(174.82-247.88) | 0.029* |
| TCH(mmol/L) | 4.66 (4.03-5.45) | 4.91 (4.31-5.62) | 0.159 |
| TP(g/L) | 65.10 (62.65-67.60) | 66.40(64.38-70.28) | 0.052 |
| INR | 1.04 (0.98-1.11) | 1.01 (0.96-1.07) | 0.019* |
| HBsAg(ng/mL) | 658.90(373.00-852.55) | 562.69(376.02-818.33) | 0.340 |

Statistically significant*. BMI body mass index, TBIL total bilirubin, ALB albumin, ALT alanine aminotransferase, AST Aspartate aminotransferase, PT prothrombin time, AFP alpha fetoprotein, Portal hypertension(mmHg), tumor size stage (cm), BCLC stage barcelona clinic liver cancer stage, PLT platelet, HGB hemoglobin, PA prealbumin, TBA total bile acid, TP total protein, TCH total cholesterol, INR international normalized ratio,HBsAg hepatitis B surface antigens, bo.PHLF: post-hepatectomy liver failure before hepatectomy, nbo.PHLF: post-hepatectomy without liver failure before hepatectomy.
