# Supplementary material for: Preoperative gut microbiota combined with machine learning for predicting post-hepatectomy liver failure in HBV-related hepatocellular carcinoma: a pilot study
Source: Front Cell Infect Microbiol. 2026 Aug 6;16:1877450. doi: 10.3389/fcimb.2026.1877450 (PMC13490075; doi:10.3389/fcimb.2026.1877450)
Supplement: Supplementary file 6 [file Table3.doc]

Table S3. Detailed Statistical Parameters of the 11 Differential Genera Identified by LefSe

| Genus | Enriched Group | LDA Score | P-value |
| --- | --- | --- | --- |
| Bacteroides | nbo.PHLF | 4.51 | <0.001 |
| Parabacteroides | nbo.PHLF | 3.56 | <0.001 |
| Dialister | nbo.PHLF | 3.01 | 0.008 |
| Erysipelotrichaceae_UCG_003 | bo.PHLF | 3.19 | 0.011 |
| Anaerostipes | bo.PHLF | 3.21 | 0.015 |
| Blautia | bo.PHLF | 4.12 | 0.003 |
| Dorea | bo.PHLF | 3.09 | 0.011 |
| Fusicatenibacter | bo.PHLF | 3.39 | 0.002 |
| Roseburia | bo.PHLF | 3.83 | 0.037 |
| Faecalibacterium | bo.PHLF | 4.43 | <0.001 |
| Subdoligranulum | bo.PHLF | 3.62 | 0.009 |
