# Supplementary material for: Preoperative gut microbiota combined with machine learning for predicting post-hepatectomy liver failure in HBV-related hepatocellular carcinoma: a pilot study
Source: Front Cell Infect Microbiol. 2026 Aug 6;16:1877450. doi: 10.3389/fcimb.2026.1877450 (PMC13490075; doi:10.3389/fcimb.2026.1877450)
Supplement: Supplementary file 7 [file Table4.doc]

Table S4 Functional alteration caused by microbial change through PICRUSt2 analysis.

| **Pathway ID** | **Altered pathway** | **P value q value** | |
| --- | --- | --- | --- |
| PWY-621 | sucrose degradation III (sucrose invertase) | 0.008 0.077 | |
| GLYCOCAT-PWY | glycogen degradation I (bacterial) | 0.024 0.166 | |
| GLYCOGENSYNTH-PWY | glycogen biosynthesis I (from ADP-D-Glucose) | 0.011 0.090 | |
| PWY0-1319 | CDP-diacylglycerol biosynthesis II | 0.040 0.217 | |
| PWY-5667 | CDP-diacylglycerol biosynthesis I | 0.040 0.217 | |
| COMPLETE-ARO-PWY | superpathway of aromatic amino acid biosynthesis | 0.045 0.221 | |
| PWY0-1296 | purine ribonucleosides degradation | 0.028 0.185 | |
| ARO-PWY | chorismate biosynthesis I | 0.046 0.221 | |
| PWY-6317 | galactose degradation I (Leloir pathway) | 0.029 0.185 | |
| P161-PWY | acetylene degradation | <0.001 0.009* | |
| PWY-5505 | L-glutamate and L-glutamine biosynthesis | 0.011 0.093 | |
| DAPLYSINESYN-PWY | L-lysine biosynthesis I | 0.002 0.024* | |
| SER-GLYSYN-PWY | superpathway of L-serine and glycine biosynthesis I | 0.033 0.201 | |
| GLYCOLYSIS | glycolysis I (from glucose 6-phosphate) | 0.023 0.164 | |
| ANAEROFRUCAT-PWY | homolactic fermentation | 0.023 0.164 | |
| ARGSYNBSUB-PWY | L-arginine biosynthesis II (acetyl cycle) | 0.001 0.017* | |
| PWY-5484 | glycolysis II (from fructose 6-phosphate) | 0.003 0.034* | |
| FASYN-ELONG-PWY | fatty acid elongation -- saturated | 0.006 0.066 | |
| ARGSYN-PWY | L-arginine biosynthesis I (via L-ornithine) | 0.002 0.026* | |
| P42-PWY | incomplete reductive TCA cycle | <0.001 0.012* | |
| PWY-7400 | L-arginine biosynthesis IV (archaebacteria) | 0.002 0.027* | |
| PWY0-1297 | superpathway of purine deoxyribonucleosides degradation | 0.007 0.071 | |
| PWY-6151 | S-adenosyl-L-methionine cycle I | 0.043 0.220 | |
| TRPSYN-PWY | L-tryptophan biosynthesis | 0.034 0.201 | |
| COBALSYN-PWY | adenosylcobalamin salvage from  cobinamide I | 0.016 0.119 | |
| PWY0-1298 | superpathway of pyrimidine deoxyribonucleosides degradation | 0.001 0.019* | |
| GLUTORN-PWY | L-ornithine biosynthesis | 0.003 0.036* | |
| PWY-6897 | thiamin salvage II | 0.035 0.203 | |
| PWY0-162 | superpathway of pyrimidine ribonucleotides de novo biosynthesis | 0.001 0.022* | |
| PWY-6519 | 8-amino-7-oxononanoate biosynthesis I | 0.010 0.090 | |
| PWY-6269 | adenosylcobalamin salvage from  cobinamide II | 0.029 0.185 | |
|  |  |  | |
| Table S4 (Continue)Functional alteration caused by microbial change through PICRUSt2 analysis. | | | |
| **Pathw****ay ID** | **Altered pat****hway** **P value q value** | | |
| PWY-5509  DENOVOPURINE2-PWY | adenosylcobalamin biosynthesis from cobyrinate a,c-diamide I  superpathway of purine nucleotides de novo biosynthesis II | 0.032 0.199  <0.001 0.008* | |
| PWY-841 | superpathway of purine nucleotides de novo  biosynthesis I | <0.001 0.006* | |
| METH-ACETATE-PWY | methanogenesis from acetate 0.007 | | 0.076 |
| PWY-6125 superpathway of guanosine nucleotides de <0.001 0.005*  novo biosynthesis II  BIOTIN-BIOSYNTHESIS biotin biosynthesis I 0.009 0.084  -PWY | | | |
| PWY-7228 | superpathway of guanosine nucleotides de  novo biosynthesis I | <0.001 0.005* | |
| PWY-7196 | superpathway of pyrimidine ribonucleosides  salvage | <0.001 0.017* | |
| P108-PWY | pyruvate fermentation to propanoate I | 0.005 0.058 | |
| PENTOSE-P-PWY | pentose phosphate pathway | <0.001 0.019* | |
| PWY0-166 | superpathway of pyrimidine  deoxyribonucleotides de novo  biosynthesis (E. coli) | <0.001 0.005* | |
| PWY-7187 | pyrimidine deoxyribonucleotides de novo  biosynthesis II | <0.001 0.005* | |
| PWY-7184 | pyrimidine deoxyribonucleotides de novo  biosynthesis I | <0.001 0.005* | |
| PWY-6901 | superpathway of glucose and xylose  degradation | 0.010 0.090 | |
| PWY-5384 | sucrose degradation IV  (sucrose phosphorylase) | 0.046 0.221 | |
| POLYISOPRENSYN-PWY | polyisoprenoid biosynthesis (E. coli) | 0.001 0.019* | |
| PWY-6700 | queuosine biosynthesis | 0.043 0.221 | |
| PWY-7197 | pyrimidine deoxyribonucleotide  phosphorylation | <0.001 0.009* | |
| PWY-6891 | thiazole biosynthesis II (Bacillus) | 0.001 0.024* | |
| PWY-7200 | superpathway of pyrimidine  deoxyribonucleoside salvage | <0.001 0.012* | |
| PWY-6507 | 4-deoxy-L-threo-hex-4-enopyranuronate  degradation | 0.039 0.216 | |
| PWY-5913 | TCA cycle VI (obligate autotrophs) | 0.042 0.220 | |
| PRPP-PWY | superpathway of histidine, purine,  and pyrimidine biosynthesis | 0.023 0.165 | |
| Table S4 (Continued)Functional alteration caused by microbial change through PICRUSt2 analysis | | | |
| **Pathway ID** | **Altered pathway** | **P value q value** | |
| PWY-7323 | superpathway of GDP-mannose-derived 0.009  O-antigen building blocks biosynthesis | | 0.087 |
| PWY-5154  PWY-6895 | L-arginine biosynthesis III (via N-acetyl-L-citrulline)  superpathway of thiamin diphosphate  biosynthesis II | 0.042 0.220  0.020 0.148 | |
| P124-PWY | Bifidobacterium shunt | 0.001 0.022* | |
| PWY-5676 | acetyl-CoA fermentation to butanoate II | 0.046 0.221 | |
| P23-PWY | reductive TCA cycle I | 0.032 0.198 | |
| PWY-1269 | CMP-3-deoxy-D-manno-octulosonate  biosynthesis I | 0.002 0.027* | |
| PWY-7211 | superpathway of pyrimidine 0.006  deoxyribonucleotides de novo  biosynthesis | | 0.068 |
| NAGLIPASYN-PWY | superpathway of (Kdo)2-lipid A biosynthesis | <0.001 0.009* | |
| PWY-7234 | inosine-5'-phosphate biosynthesis III | 0.036 0.205 | |
| FUCCAT-PWY | fucose degradation | 0.003 0.036* | |
| HISDEG-PWY | L-histidine degradation I | <0.001 0.017* | |
| COLANSYN-PWY | colanic acid building blocks biosynthesis | 0.001 0.019* | |
| PWY-7315 | dTDP-N-acetylthomosamine biosynthesis | 0.008 0.076 | |
| GLUCUROCAT-PWY | superpathway of hexuronide and hexuronate degradation | 0.003 0.040* | |
| PWY-6467 | Kdo transfer to lipid IVA III (Chlamydia) | 0.002 0.024* | |
| P122-PWY | heterolactic fermentation | 0.001 0.024* | |
| GALACT-GLUCUROCAT  -PWY | superpathway of hexuronide and hexuronate degradation | 0.042 0.220 | |
| PWY-7456 | mannan degradation | <0.001 0.009* | |
| PWY-4984 | urea cycle | <0.001 0.005* | |
| PYRIDOXSYN-PWY | pyridoxal 5'-phosphate biosynthesis I | 0.038 0.215 | |
| PWY-6572 | chondroitin sulfate degradation I (bacterial) | 0.027 0.181 | |
| P163-PWY | L-lysine fermentation to acetate and butanoate | 0.010 0.089 | |
| PWY-6590 | superpathway of Clostridium acetobutylicum acidogenic fermentation | <0.001 0.011* | |

PWY-7210	pyrimidine deoxyribonucleotides	0.017	0.127

| CENTFERM-PWY | biosynthesis from CTP  pyruvate fermentation to butanoate | <0.001 0.009* |
| --- | --- | --- |
| PWY-7198 | pyrimidine deoxyribonucleotides de  novo biosynthesis IV | 0.025 0.174 |
| Table S4 (Continued)Functional alteration caused by microbial change through PICRUSt2 analysis. | | |
| **Pathway ID** | **Altered pathway** | **P value q value** |
| P381-PWY | adenosylcobalamin biosynthesis II  (late cobalt incorporation) | 0.026 0.176 |
| P162-PWY | L-glutamate degradation V  (via hydroxyglutarate) | 0.011 0.093 |
| PWY-5920 | superpathway of heme biosynthesis  from glycine | 0.015 0.119 |
| PWY-7332 | superpathway of UDP-N  -acetylglucosamine-derived  O-antigen building blocks biosynthesis | 0.009 0.085 |
| PWY-6396 | superpathway of 2,3-butanediol biosynthesis | 0.034 0.202 |
| P125-PWY | superpathway of (R,R)-butanediol  biosynthesis | 0.013 0.107 |
| PWY-5823 | superpathway of CDP-glucose-derived  O-antigen building blocks biosynthesis | 0.039 0.216 |
| PWY-7090 | UDP-2,3-diacetamido-2,3-dideoxy-&alpha;-D-mannuronate biosynthesis | 0.006 0.066 |
| PWY-4722 | creatinine degradation II | 0.012 0.095 |
| PWY-3941 | beta;-alanine biosynthesis II | 0.048 0.230 |

Statistically significant (q value)*.
