# Supplementary material for: Preoperative gut microbiota combined with machine learning for predicting post-hepatectomy liver failure in HBV-related hepatocellular carcinoma: a pilot study
Source: Front Cell Infect Microbiol. 2026 Aug 6;16:1877450. doi: 10.3389/fcimb.2026.1877450 (PMC13490075; doi:10.3389/fcimb.2026.1877450)
Supplement: Supplementary file 9 [file Table7.doc]

Table S7.Table showing the diagnosis performance of 3 types of machine-learing models using characteristic ASVs methods in training set.

| Performance Dimension & Models | GBM | RANDOMFOREST | XGBOOST |
| --- | --- | --- | --- |
| **Global Discrimination** |  |  |  |
| Accuracy (%) | 78.77 (74.37–84.85) | 97.21 (95.78–98.32) | 81.01 (78.03–83.11) |
| AUC (%) | 86.44 (83.06–91.31) | 99.83 (99.63–99.91) | 90.22 (88.39–94.45) |
| **PHLF Identification**  **(Rule-In Capacity)** |  |  |  |
| Sensitivity (%) | 76.60 (67.67–89.22) | 96.77 (93.45–98.47) | 80.85 (74.75–94.72) |
| PPV (%) | 57.14 (48.11–65.17) | 95.24 (89.98–97.79) | 60.32 (51.61–68.80) |
| F1-positive(Standard F1-score) (%) | 65.45 (59.25–74.29) | 96.00 (93.49–97.73) | 69.09 (62.39–74.04) |
| ***Non-PHLF Exclusion***  ***(Rule-Out Capacity)***  ***— Core Thesis*** |  |  |  |
| Specificity (%) | 79.55 (73.36–84.91) | 97.44 (95.04–99.04) | 81.06 (77.72–85.50) |
| NPV (%) | 90.52 (87.26–96.13) | 98.28 (96.90–99.17) | 92.24 (88.17–97.77) |
| **F1-negative(Exclusionary F1-score) (%)** | **84.68 (Derived)** | **97.86 (Derived)** | **86.29 (Derived)** |

Note: Data are presented as value (95% confidence interval). F1-positive refers to the standard F1-score targeted at the positive class (PHLF). F1-negative represents the exclusionary F1-score targeted at the non-PHLF class, calculated as F-1negative = 2 *(Specificity*NPV)/(Specificity + NPV);

PPV : Positive Predictive Value ;

NPV : Negative Predictive Value;

PHLF: post-hepatectomy liver failure;

non-.PHLF: post-hepatectomy without liver failure.
