# Supplementary material for: Preoperative gut microbiota combined with machine learning for predicting post-hepatectomy liver failure in HBV-related hepatocellular carcinoma: a pilot study
Source: Front Cell Infect Microbiol. 2026 Aug 6;16:1877450. doi: 10.3389/fcimb.2026.1877450 (PMC13490075; doi:10.3389/fcimb.2026.1877450)
Supplement: Supplementary file 10 [file Table8.doc]

Table S8.Table showing the diagnosis performance of 3 types of machine-learing models using characteristic ASVs methods in internal validation set.

| Performance Dimension & Models | GBM | | | RANDOMFOREST | XGBOOST |
| --- | --- | --- | --- | --- | --- |
| **Global Discrimination** |  | | |  |  |
| Accuracy (%) | 75.00(55.94–86.80) | | | 75.00 (62.50–86.80) | 78.12(75.00–89.22) |
| AUC (%) | 78.35 (51.33–89.18) | | | 77.49 (57.01–84.19) | 81.60(72.71–93.87) |
| **PHLF Identification** |  | | |  |  |
| **(Rule-In Capacity)** |  | | |  |  |
| Sensitivity (%) | 66.67 (41.00–96.79) | | | 63.64 (44.83–97.95) | 70.00(47.57–91.25) |
| PPV (%) | 54.55 (19.25–77.00) | | | 63.64 (34.02–85.47) | 63.64(55.13–87.30) |
| F1-positive(Standard F1-score) (%) | 60.00 (25.79–81.01) | | | 63.64 (41.00–82.26) | 66.67(52.25–83.88) |
| ***Non-PHLF Exclusion*** |  | | |  |  |
| ***(Rule-Out Capacity)*** |  | | |  |  |
| ***— Core Thesis*** |  | | |  |  |
| Specificity (%) | 78.26 (58.99–85.97) | | | 80.95 (62.76–93.27) | 81.82(68.81–94.23) |
| NPV (%) | 85.71 (77.70–97.86) | | | 80.95 (72.16–98.82) | 85.71(77.27–92.08) |
| **F1-negative(Exclusionary** | **81.82 (Derived)** | | | **80.95 (Derived)** | **83.72 (Derived)** |
| **F1-score) (%)** | |  | |  |  |
|  | | |  |  |  |

Note: Data are presented as value (95% confidence interval). F1-positive refers to the standard F1-score targeted at the positive class (PHLF). F1-negative represents the exclusionary F1-score targeted at the non-PHLF class, calculated as F1-negative = 2 *(Specificity*NPV)/(Specificity + NPV);

PPV : Positive Predictive Value ;

NPV : Negative Predictive Value;

PHLF: post-hepatectomy liver failure;

non-.PHLF: post-hepatectomy without liver failure.
