# Supplementary material for: Preoperative gut microbiota combined with machine learning for predicting post-hepatectomy liver failure in HBV-related hepatocellular carcinoma: a pilot study
Source: Front Cell Infect Microbiol. 2026 Aug 6;16:1877450. doi: 10.3389/fcimb.2026.1877450 (PMC13490075; doi:10.3389/fcimb.2026.1877450)
Supplement: Supplementary file 11 [file Table9.doc]

Table S9.Table showing the diagnosis performance of 3 types of machine-learing models using characteristic ASVs methods in independent external validation set.

| Performance Dimension & Models | GBM | RANDOMFOREST | XGBOOST |
| --- | --- | --- | --- |
| **Global Discrimination** |  |  |  |
| Accuracy (%) | 63.64 (42.95–85.34) | 72.73 (60.11–77.27) | 72.73 (59.09–81.82) |
| AUC (%) | 63.54 (38.14–76.95) | 71.88 (52.45–82.52) | 78.12 (59.67–98.41) |
| **PHLF Identification** |  |  |  |
| **(Rule-In Capacity)** |  |  |  |
| Sensitivity (%) | 25.00 (0.00–48.57) | 50.00 (15.31–79.58) | 50.00 (21.93–70.98) |
| PPV (%) | 16.67 (0.00–36.67) | 50.00 (26.87–59.00) | 50.00 (21.35–95.50) |
| F1-positive(StandardF1-score) (%) | 20.00 (0.00–38.67) | 50.00 (21.59–64.17) | 50.00 (23.09–64.52) |
| ***Non-PHLF Exclusion*** |  |  |  |
| ***(Rule-Out Capacity)*** |  |  |  |
| ***— Core Thesis*** |  |  |  |
| Specificity (%) | 72.22 (59.34–88.77) | 81.25 (63.00–87.31) | 81.25 (70.78–98.68) |
| NPV (%) | 81.25 (51.76–89.36) | 81.25 (63.95–92.73) | 81.25 (54.63–90.91) |
| **F1-negative(Exclusionary** | **76.47 (Derived)** | **81.25 (Derived)** | **81.25 (Derived)** |
| **F1-score) (%)** |  |  |  |

Note: Data are presented as value (95% confidence interval). F1-positive refers to the standard F1-score targeted at the positive class (PHLF). F1-negative represents the exclusionary F1-score targeted at the non-PHLF class, calculated as F1negative = 2 *(Specificity*NPV)/(Specificity + NPV);

PPV : Positive Predictive Value ;

NPV : Negative Predictive Value;

PHLF: post-hepatectomy liver failure;

non-.PHLF: post-hepatectomy without liver failure.
